# Slow and Fast Evolving Endosymbiont Lineages: Positive Correlation between the Rates of Synonymous and Non-Synonymous Substitution

**DOI:** 10.3389/fmicb.2015.01279

**Published:** 2015-11-13

**Authors:** Francisco J. Silva, Diego Santos-Garcia

**Affiliations:** ^1^Institut Cavanilles de Biodiversitat i Biologia Evolutiva, Universitat de ValènciaValència, Spain; ^2^Unidad Mixta de Investigación en Genómica y Salud, Fundación para el Fomento de la Investigación Sanitaria y Biomédica de la Comunitat Valenciana, Salud Pública/Institut Cavanilles, Universitat de ValènciaValència, Spain; ^3^Department of Entomology, Hebrew University of JerusalemRehovot, Israel

**Keywords:** endosymbiosis, evolutionary rate, nucleotide substitution, generation time, DNA replication, DNA repair

## Abstract

The availability of complete genome sequences of bacterial endosymbionts with strict vertical transmission to the host progeny opens the possibility to estimate molecular evolutionary rates in different lineages and understand the main biological mechanisms influencing these rates. We have compared the rates of evolution for non-synonymous and synonymous substitutions in nine bacterial endosymbiont lineages, belonging to four clades (*Baumannia*, *Blochmannia*, *Portiera*, and *Sulcia*). The main results are the observation of a positive correlation between both rates with differences among lineages of up to three orders of magnitude and that the substitution rates decrease over long endosymbioses. To explain these results we propose three mechanisms. The first, variations in the efficiencies of DNA replication and DNA repair systems, is unable to explain most of the observed differences. The second, variations in the generation time among bacterial lineages, would be based on the accumulation of fewer DNA replication errors per unit time in organisms with longer generation times. The third, a potential control of the endosymbiont DNA replication and repair systems through the transfer of nuclear-encoded proteins, could explain the lower rates in long-term obligate endosymbionts. Because the preservation of the genomic integrity of the harbored obligate endosymbiont would be advantageous for the insect host, biological mechanisms producing a general reduction in the rates of nucleotide substitution per unit of time would be a target for natural selection.

## Introduction

The main problem to estimate and compare the rates of sequence evolution among bacterial lineages is the difficulty to obtain the times of divergence of the compared species and strains. Although for culturable bacteria, in very short periods of time, an experimental approach in laboratory may be applied (Lenski et al., 2003), the estimations of the rates of nucleotide substitution over long periods of time are intractable. However, there is one exception: host-associated bacteria. Because these organisms coevolve with eukaryotic lineages, the times of divergence of the hosts may be estimated with divergence dating methods using calibration points based on fossil records. Once estimated, host divergence times may be used to obtain the rates of nucleotide substitution in the endosymbionts, provided that transmission from host to offspring is strictly vertical. In insects, many endosymbiont lineages with strict vertical transmission for millions of years have been described (Baumann, 2005; Moran et al., 2008; Moya et al., 2008). They are ideal subjects to be analyzed although, until now, only some studies using rough estimations of the times of divergence among insect taxa have been performed. The use of methods for temporal calibration of molecular phylogenies permits the estimation of the rates of molecular evolution within any endosymbiotic clade with strictly vertical transmission.

## Rates Of Gene Sequence Evolution In Bacterial Endosymbionts

The first comparisons of the rates of gene sequence evolution between endosymbionts and free living microorganisms were mainly focused on rRNA genes. It was observed through relative rate tests the significant acceleration of the 16S rRNA genes of the primary endosymbionts of aphids (*Buchnera aphidicola*), mealybugs, whiteflies, and tse-tse flies in comparison with close free-living relatives (Moran, 1996). This kind of fast evolution was later reported for sulfur-oxidizing bacteria (Peek et al., 1998), fungi (Lutzoni and Pagel, 1997), or fungi and bacteria (Woolfit and Bromham, 2003). These comparative analyses were extended to a few coding genes. For example, the number of non-synonymous substitutions per site (dN) was higher in several *Buchnera* genes than in their corresponding orthologs in enterics (Moran, 1996; Clark et al., 1999). However, the differences in the number of synonymous substitutions per site (dS) between the endosymbiont (*Buchnera*) and the free-living relative were small (Clark et al., 1999).

Later, the comparative analyses were extended to different endosymbiont lineages, among then and with their free-living relatives, and the estimated dS were weighted by estimations of the times of divergence. In this case, large differences were observed for the dS/t (dS/time) of the genes *gidA* and *groEL* with estimations of around 1 × 10^-7^, 1 × 10^-8^ and 1 × 10^-9^ synonymous substitutions per site per year for “*Candidatus* Blochmannia” (endosymbiont of carpenter ants, hereafter *Blochmannia*), *Buchnera* and free-living enterics, respectively (Degnan et al., 2004). Rates of evolution for non-functional DNA sequences were also estimated for *Buchnera* and *Blochmannia* at 4.3 × 10^-9^ and 1.5 × 10^-8^, respectively (Gomez-Valero et al., 2007, 2008).

Although the analysis of a reduced set of genes may give clues about the rates of molecular evolution, the availability of the complete endosymbiont genomes may contribute to get a whole view of the rates of evolution in each symbiont lineage, not affected by the random or selective processes of each gene.

Recently, two analyses involving the estimation of dN and dS (or dN/t and dS/t) at genomic scale and the comparison among different endosymbiont lineages have been performed (Bennett et al., 2014; Santos-Garcia et al., 2015). In the first study, two obligate endosymbionts (“*Candidatus* Sulcia muelleri” and “*Candidatus* Baumannia cicadellinicola,” hereafter *Sulcia* and *Baumannia*, respectively) of two sharpshooters species (Hemiptera:Cicadellidae:Cicadellinae) of the tribes Proconiini (*Homalodisca vitripennis*) and Cicadellini (*Graphocephala atropunctata*) were analyzed. For both symbionts, the start of the symbiosis took place before the divergence of both insect lineages, but while *Sulcia* has a long-term coevolutionary history with Auchenorrhyncha, the relation of *Baumannia* with sharpshooters is much more recent, because it replaced another ancestral symbiont in the subfamily Cicadellinae, probably as a consequence of a diet change from phloem to xylem (Bennett and Moran, 2013). Because the same divergence time may be applied to the two *Baumannia* strains and the two *Sulcia* strains, dN and dS were compared in spite of the absence of a concrete time of divergence. The average gene dN was 28-fold higher in *Baumannia* than in *Sulcia*, while the differences in average gene dS were even higher (89-fold) (Bennett et al., 2014). This clearly differentiates *Baumannia* and *Sulcia* as fast and slow evolving endosymbionts.

In the second study, the rates of dN/t and dS/t were estimated in four lineages of “*Candidatus* Portiera aleyrodidarum” (hereafter *Portiera*), the endosymbiont of whiteflies (Santos-Garcia et al., 2015). The divergence times of the lineages were estimated with the program BEAST (Bouckaert et al., 2014), sequence datasets from the endosymbionts and mitochondria and calibrators for the nodes of divergence of the two whitefly subfamilies (125–135 My) and of aphids and whiteflies (250–278 My). The results showed significant differences in the rate of evolution of the lineages, and an interesting correlation between dN/t and dS/t, with *Portiera* from *Bemisia tabaci* evolving for both rates fivefold higher than the slow-evolving *Portiera* from *Aleurodicus floccissimus*. High dN and dS values in *Portiera* from *B. tabaci* had been previously reported when compared to *Portiera* from *Trialeurodes vaporariorum* (Sloan and Moran, 2013).

With the aim to compare at genomic scale the rates of nucleotide substitution in several endosymbiont lineages, we selected two long-term and two relatively recent endosymbiont clades. *Sulcia* and *Portiera* were the long-term clades. The infection of *Sulcia* took place 260–280 My ago (Moran et al., 2005), while in *Portiera*, the original infection probably took place in the ancestors of psyllids and whiteflies (>250 My ago), leading with time to the endosymbiotic genera “*Candidatus* Carsonella” and *Portiera*, respectively (Santos-Garcia et al., 2014). *Baumannia*, whose infection took place at least 50 My ago (Koga and Moran, 2014) and *Blochmannia*, whose infection of the ant tribe Camponotini took placed around 40 Mya (Williams and Wernegreen, 2015) were the short-term clades.

**FIGURE 1:**
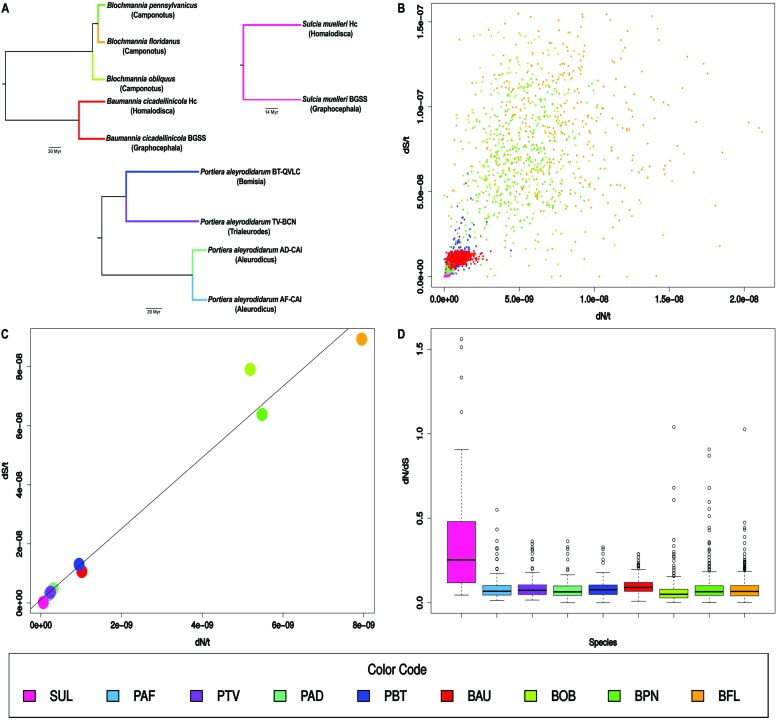
**Sequence evolution in bacterial endosymbiont lineages.** **(A)** Tree topologies for the nine analyzed lineages (host genus between parentheses) were based on concatenated protein alignments (Bennett et al., 2014; Santos-Garcia et al., 2015; Bennett and Moran, 2013; Williams and Wernegreen, 2015). Branch colors mark the lineages. Notice that the analyses of *Sulcia* and *Baumannia* were not performed in a single branch. **(B)** Scatter plot of dN/t *vs.* dS/t. Each point is a gene. **(C)** Scatter plot of dN/t *vs.* dS/t. Each point is the average gene value for each lineage. A regression line is displayed. **(D)** Box plot for the dN/dS values of the genes in each lineage. Color codes and abbreviations are shown at the end. They were ordered according to dN/t from smallest to largest. Abbreviations: SUL, *Sulcia*; PAF, *Portiera A. floccissimus*; PTV, *Portiera T. vaporariorum*; PAD, *Portiera A. dispersus*; PBT, *Portiera B. tabaci*; BAU, *Baumannia*; BOB, *B. obliquus*; BPN, *B. pennsylvanicus*; and BFL, *B. floridanus*.

Only the dN/t and dS/t values from four *Portiera* lineages were directly obtained from a previous report (Santos-Garcia et al., 2015), while values for the remaining lineages were estimated for this work. *Baumannia* (BAU) and *Sulcia* (SUL) dN and dS gene estimations were obtained from the Supplementary Material of a previous publication (Bennett et al., 2014). In *Sulcia*, only the gene set retained for average estimations by Bennett et al. (2014), excluding those with zero dN or dS values and in *Baumannia*, the unsaturated genes. Because dN and dS were pairwise estimations, we obtained the dN/t and dS/t values corresponding to the substitutions in the two analyzed branches (**Figure 1A**) using twice the time of divergence of the lineages. We used a time of divergence between *H. vitripennis* and *G. atropunctata* of 66.2 My, an average of the estimations of 69 My and 63.4 My obtained for their *Sulcia* and *Baumannia* endosymbionts (Szklarzewicz et al., 2015), because the value should be the same if both symbionts have strict vertical transmission.

Finally, we estimated the dN and dS gene values from the gene sequences reported in the genomes of *Blochmannia floridanus*, endosymbiont of *Camponotus floridanu*s (Gil et al., 2003), *Blochmannia pennsylvanicus*, endosymbiont of *Camponotus pennsylvanicus* (Degnan et al., 2005) and the more distant relative, *Blochmannia obliquus* endosymbiont of *Colobopsis obliquus* (Williams and Wernegreen, 2015). To obtain these values, genomes of these species and *Baummania* as outgroup were obtained and their proteins classified in orthologous groups with OrthoMCL (Supplementary Material, Li et al., 2003). Then, codon alignments were obtained and codeML (Yang, 2007) was used to obtain the dN and dS values as previously described (Santos-Garcia et al., 2015). Only those genes with no zero dN or dS values and dS values less than four were selected for plotting and other analyses (the data files and the R markdown file can be found in Supplementary material). Three lineages were considered, the one leading to *B. obliquus* (BOB), whose period of evolution was estimated at 32.6 My (Degnan et al., 2004) and the two lineages leading to *B. floridanus* (BFL) and *B pennsylvanicus* (BPN), with a period of evolution of 18 My each (Degnan et al., 2004).

Gene dN/t and dS/t values in the nine lineages (**Figure 1A**) were plotted (**Figure 1B**). A significant positive correlation (*r*^2^ = 0.54) (*p*-value <0.001) was observed under the linear regression model When the average values for dN/t and dS/t in each lineage were plotted (**Figure 1C**), a positive correlation (*r*^2^ = 0.98) was more clearly observed. *Sulcia* was the slowest evolving lineage followed by three out of the four *Portiera* lineages. *Portiera* from *B. tabaci* and *Baumannia* evolved with intermediate values. Finally, the rates of evolution of the three *Blochmannia* lineages were very high with the values of *B. floridanus* around 140-fold and 600-fold higher than *Sulcia* for dN/t and dS/t, respectively. On the contrary, the average dN/dS ratios were similar in most of the lineages, except in *Sulcia* which showed a value of around 0.30 (**Figure 1D**).

Although only a limited number of lineages have been analyzed, the main general conclusion that we can extract is that the evolution of the gene sequences in bacterial endosymbionts are triggered by mechanisms affecting both substitution rates. The second conclusion is that the long-term endosymbiotic clades (*Sulcia* and *Portiera*) evolve more slowly than the recent ones (*Baumannia* and *Blochmannia*). The exception is one of the lineages of *Portiera*, but it may be explained by the genome instability associated with this lineage (Sloan and Moran, 2012, 2013).

## Evolutionary Processes Explaining The Correlation Between dN/t And dS/t In Bacterial Endosymbionts

The selection of four bacterial endosymbiotic clades for the analysis of the rates of coding gene evolution at genomic scale shows very large differences, in the range of three orders of magnitude between the slowest and the fastest evolving lineages. To explain this observation, we need to understand how endosymbiotic lifestyle may alter fundamental evolutionary processes such as mutation, selection, genetic drift, and recombination, and the profound consequences on the rates of nucleotide substitution (Wernegreen, 2015).

The heterogeneity of the gene dN/dS ratios within a specific lineage may be conducted basically by natural selection. Positive selection may favor non-synonymous changes in some positions as an adaptation to changes in the intracellular environment, or purifying selection may remove a larger proportion of non-synonymous changes in some genes than in others. But, neither of these conditions affect both synonymous and non-synonymous rates (**Figure 1C**). To explain this plot we need to consider mechanisms that alter simultaneously the rates of synonymous and non-synonymous substitutions. Both rates will not be the same because natural selection will favor the removal of most of the non-synonymous substitutions avoiding their transfer to the next generation but, in theory, it will have almost no effect over the synonymous ones, by its neutrality for most of the changes. However, natural selection may affect both rates simultaneously, if its action consists of selecting general mechanisms that either increase or decrease the mutation at any position of the DNA sequence. In this regard, we might predict that mechanisms lowering mutation rates would be favored in primary endosymbionts (Wernegreen, 2015).

Which are, thus, the mechanisms that can explain the differences of three orders of magnitude in the rates of the four analyzed symbiont clades? We propose three mechanisms, with the understanding that they are not mutually exclusive.

The first possible mechanism we considered is the efficiency of DNA replication and repair systems. The lower the efficiency, the higher the rates of mutation and substitution. The overall efficiency of these two systems involves many components. Not only is it difficult to analyze these components, but to determine how they are combined to generate an overall efficiency. Many mechanisms for DNA repair have been described in living beings including direct reversal, base excision repair, nucleotide excision repair and recombination (Sancar et al., 2004). Their efficiencies depend on many factors, such as, for example, the variety, concentration, error rate and activity of DNA replication and repair enzymes. The number of genes encoding proteins involved in DNA replication and DNA repair is one of these components (**Table 1**), although the observation of a lower number of genes in this category does not mean automatically a high substitution rate since it may be compensated by better performances of other components. Only within one clade, we can observe some changes that may be associated with an increase of the mutation rate based on the loss of DNA repair and replication enzymes. This is the case of *Portiera* from *B. tabaci* B and Q biotypes (Santos-Garcia et al., 2012; Sloan and Moran, 2012) which has lost a large number of genes for DNA replication and repair comparing with the last common ancestor of *Portiera* strains (Santos-Garcia et al., 2015). However, when we compared the repertoires for these functional categories among the nine analyzed genomes, we did not find a general relationship between rates of substitution and number of genes in these categories and, in fact, the genera with the largest number of genes in these categories (*Baumannia* and *Blochmannia*), are those displaying the largest substitution rates (**Table 1**).

**Table 1:** Number of genes in the DNA replication and repair category and rates of nucleotide substitution (average values).

	SUL	PAF	PTV	PAD	PBT	BAU	BOB	BPN	BFL
Gene number	6	13	13	13	5	26	18	18	18
dS/t	1.5 × 10^-10^	2.9 × 10^-09^	3.6 × 10^-09^	4.8 × 10^-09^	1.3 × 10^-08^	1.1 × 10^-08^	7.9 × 10^-08^	6.4 × 10^-08^	8.9 × 10^-08^
dN/t	5.8 × 10^-11^	2.0 × 10^-10^	2.4 × 10^-10^	3.1 × 10^-10^	9.5 × 10^-10^	1.0 × 10^-09^	5.2 × 10^-09^	5.6 × 10^-09^	7.9 × 10^-09^


*See abbreviations in **Figure 1**.*

From the first theoretical studies, generation time was considered as one of the main factors contributing to explain the variability of the rates of molecular evolution among lineages. The generation time hypothesis was based on the idea that species with shorter generation times would have larger rates of mutations per year considering that larger numbers of DNA replications per unit of time generate larger numbers of mutations. Initially, the effect of generation time in vertebrates was only clearly observed in synonymous rates (Ohta, 1993). The difficulties to detect this effect in non-synonymous rates were explained by the fact that species with larger generation times also tend to have smaller effective population sizes and both factors are compensated. Effects of generation time on the molecular evolutionary rates have also been described in other taxonomic groups such as invertebrates (Thomas et al., 2010) and, in this case, the effect was evident for both synonymous and non-synonymous rates.

In spite of the difficulties of study the effect of generation time in bacteria, it has been recently reported that, in Firmicutes, the DNA mutation and the protein evolutionary rates are smaller in spore-forming lineages than in non-spore-forming lineages (Weller and Wu, 2015). Because, it is expected that the generation time is longer in spore-forming lineages, the authors indicate that their results support the effect of generation time also in bacteria.

In our analysis with endosymbionts, generation time may explain the observed differences, although no information over the number of DNA replications per unit of time may be easily estimated. However, the differences between *B. floridanus* and *B. pennsylvanicus* could be explained by the behavior of their hosts (*Camponotus floridanus* and *Camponotus pennsylvanicus*) with the former displaying year-round activity and the latter winter dormancy (Degnan et al., 2005). These host traits would probably affect to the number of bacterial generations and the rates of substitution per unit of time.

If the large differences among *Sulcia* and *Portiera* by one way and *Baumannia* and *Blochmannia* by the other are related to generation time, we cannot discard that it was natural selection, which selected that the former replicate their genomes with slower rates per unit of time than the latter. By this way, the degenerative effects associated with intracellular life will be weakened avoiding its replacement by a novel and more efficient endosymbiont (Santos-Garcia et al., 2015).

The third proposed mechanism is based on the recent demonstration that an insect protein was transferred to an endosymbiont, in the aphid endosymbiont *B. aphidicola* (Nakabachi et al., 2014) and the suggestion that the lack of a specific aminoacyl-tRNA synthetase in the endosymbiont “*Candidatus* Evansia muelleri” may be compensated by the import of the insect nuclear-encoded protein (Santos-Garcia et al., 2014). After, a long-co-evolutionary relation, some insects would have taken power on their long-term endosymbionts, and to avoid their destruction, a mechanism of targeting some proteins inside the endosymbiont cell would have been developed. Some of the targeted proteins may be DNA repair enzymes, which would be able to remove many of the new mutations generated in the endosymbiont genome. Such a kind of mechanism for general decrease of the rates of mutation would have been selected and would explain why some endosymbiont lineages evolve with average rates for synonymous changes as small as 1 × 10^-10^.

## Conclusion

The huge variation among the rates of synonymous substitution among lineages of endosymbiotic bacteria cannot be explained by differences in the genetic arsenal required to avoid the production of mutations. The fact that this variation also affects to the non-synonymous rates requires that one or several general mechanisms decreasing the number of substitution by unit of time were involved. The generation time effect could explain this variability but, it is not necessary that the hosts display differences in their generation times. A slow rate of DNA replication and cell division in the endosymbionts will be sufficient to reduce the number of mutations per unit of time. The idea that the hosts may take the control of the endosymbiont rate of DNA replication or decrease the number of mutations by the transport of nuclear-encoded proteins within the endosymbiotic bacterial cell should be explored.

## Author Contributions

FS conceived research. FS and DS-G designed the analyses. DS-G performed the experiments. FS wrote the manuscript with inputs from DS-G. Both authors read and approved the final manuscript.

## Conflict of Interest Statement

The authors declare that the research was conducted in the absence of any commercial or financial relationships that could be construed as a potential conflict of interest.
