# Supplementary material for: Slow and Fast Evolving Endosymbiont Lineages: Positive Correlation between the Rates of Synonymous and Non-Synonymous Substitution
Source: Front Microbiol. 2015 Nov 13;6:1279. doi: 10.3389/fmicb.2015.01279 (PMC4643148; doi:10.3389/fmicb.2015.01279)
Supplement: Supplementary file 1 [file Data_Sheet_1.ZIP › Supplementary Material_Silva-Santos.docx]

Supplementary Material

Slow and fast evolving endosymbiont lineages: positive correlation between the rates of synonymous and nonsynonymous substitution

Francisco J. Silva* and Diego Santos-Garcia

*** Correspondence:** Francisco J. Silva: francisco.silva@uv.es

# Supplementary File

Supplemetary_File.zip includes several files:

Markdown.pdf: R commands used for the statistics

Blochmannia_Baumannia_OrthoMCL_clusters.txt: The result of applying OrthoMCL to *Baumannia* and *Blochmannia* proteomes. These clusters were used for gene alignments prior to estimate dN and dS in *Blochmannia* lineages.

Four files (Baumannia_dNdS.txt, Portieras_dNdS.txt, RAW_dNdS_data_Blochmannia.txt, Sulcia_dNdS.txt): contain data used for dN and dS analyses
