# Supplementary material for: Slow and Fast Evolving Endosymbiont Lineages: Positive Correlation between the Rates of Synonymous and Non-Synonymous Substitution
Source: Front Microbiol. 2015 Nov 13;6:1279. doi: 10.3389/fmicb.2015.01279 (PMC4643148; doi:10.3389/fmicb.2015.01279)
Supplement: Supplementary file 1 [file Data_Sheet_1.ZIP › Supplemetary_File.zip!/markdown.pdf]

Portiera, Baumannia and Blochmannia dN/dS analysis

Santos-Garcia D., Silva F.J.

Firts, install and load required packages:

install.packages("psych") install.packages("sm") install.packages("car") install.packages("lsr") install.packages("coin")

```
library(psych)
library(graphics)
library(sm)
```

```
## Package 'sm', version 2.2-5.4: type help(sm) for summary information
```

```
library(car)
```

```
##
## Attaching package: 'car'
##
## The following object is masked from 'package:psych':
##
##     logit
```

```
library(lsr)
library(coin)
```

```
## Loading required package: survival
```

Read the raw values of dN and dS calculated with codeML and store in differnet variables. Change to the folder were the data are stored:

```
setwd("/home/diego/Dropbox/Paper_dNdS_Paco/Paco/compliantFasta/Data")

#Read data:
Portiera_RAW <- read.table(file="Portieras_dNdS.tab",header=T,sep="\t")
Blochmannia_RAW <- read.table(file="RAW_dNdS_data_Blochmannia.tab",header=T,sep="\t")
Baumannia_RAW <- read.table(file="Baumannia_dNdS.tab",header=T,sep="\t")
Sulcia_RAW <- read.table(file="Sulcia_dNdS.tab",header=T,sep="\t")
```

Create one dataframe for organism:

```
PAQ <- Portiera_RAW[,c(4,5)]
PAD <- Portiera_RAW[,c(2,3)]
PAF <- Portiera_RAW[,c(6,7)]
PTV <- Portiera_RAW[,c(8,9)]
BlOb <- Blochmannia_RAW[c(4,5)]
BlFl <- Blochmannia_RAW[c(2,3)]
BlPe <- Blochmannia_RAW[c(6,7)]
Bau <- Baumannia_RAW[c(2,3)]
Sul <- Sulcia_RAW[c(2,3)]
```

Blochmannia data cleaning:

```
#Discard dN or dS equal to 0:
BlOb <- subset(BlOb,BlOb$dS_BoOb >0 & BlOb$dN_BoOb >0)
BlFl <- subset(BlFl,BlFl$dS_BoFl >0 & BlFl$dN_BoFl >0)
BlPe <- subset(BlPe,BlPe$dS_BoPe >0 & BlPe$dN_BoPe >0)
boxplot(BlOb$dS,BlPe$dS,BlFl$dS)
```

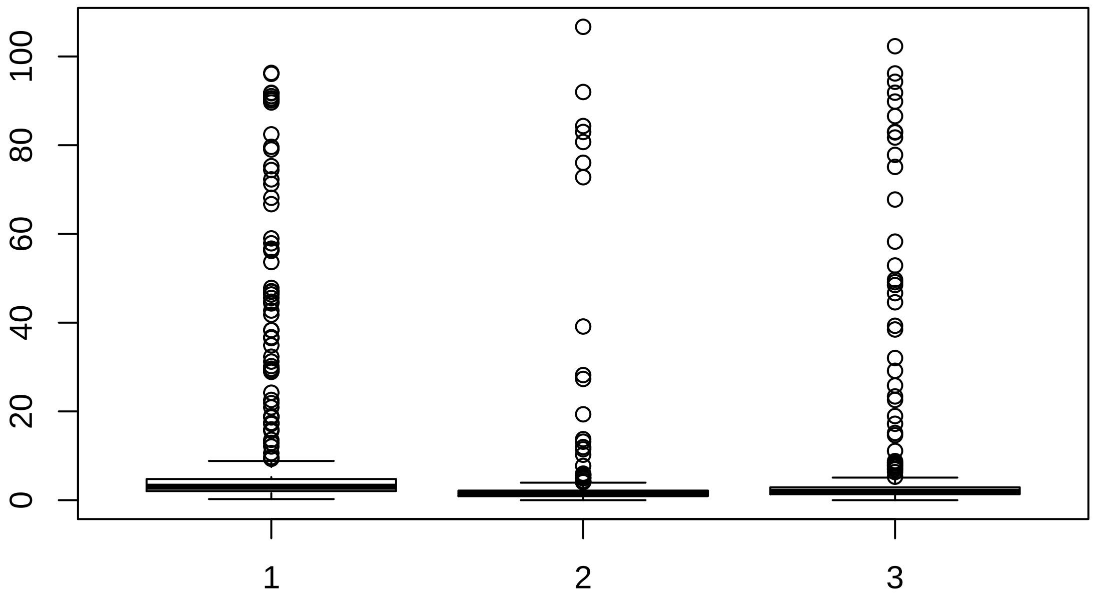


```
summary(BlOb)
```

```
##     dN_BoOb           dS_BoOb
##  Min.   :0.0218   Min.   : 0.2381
##  1st Qu.:0.1188   1st Qu.: 2.0407
##  Median :0.1739   Median : 3.0326
##  Mean   :0.1799   Mean   : 9.4847
##  3rd Qu.:0.2263   3rd Qu.: 4.7619
##  Max.   :0.6908   Max.   :96.2942
```

```
summary(BlPe)
```

```
##     dN_BoPe            dS_BoPe
##  Min.   :0.00360   Min.   :  0.0001
##  1st Qu.:0.07107   1st Qu.:  0.9116
##  Median :0.10155   Median :  1.3729
##  Mean   :0.10676   Mean   :  3.4337
##  3rd Qu.:0.13210   3rd Qu.:  2.1532
##  Max.   :0.63790   Max.   :106.6744
```

```
summary(BlFl)
```

```
##     dN_BoFl            dS_BoFl
##  Min.   :0.00450   Min.   :  0.0001
```

```
##  1st Qu.:0.09767   1st Qu.:  1.3402
##  Median :0.14150   Median :  1.8910
##  Mean   :0.14728   Mean   :  6.0430
##  3rd Qu.:0.18210   3rd Qu.:  2.8799
##  Max.   :0.50790   Max.   :102.3096
```

```
#Remove extreme dS values (putative artifacts)
BlOb <- subset(BlOb,BlOb$dS_BoOb < 4.7)
BlFl <- subset(BlFl,BlFl$dS_BoFl < 2.8)
BlPe <- subset(BlPe,BlPe$dS_BoPe < 2.1)
boxplot(BlOb$dS,BlPe$dS,BlFl$dS)
```

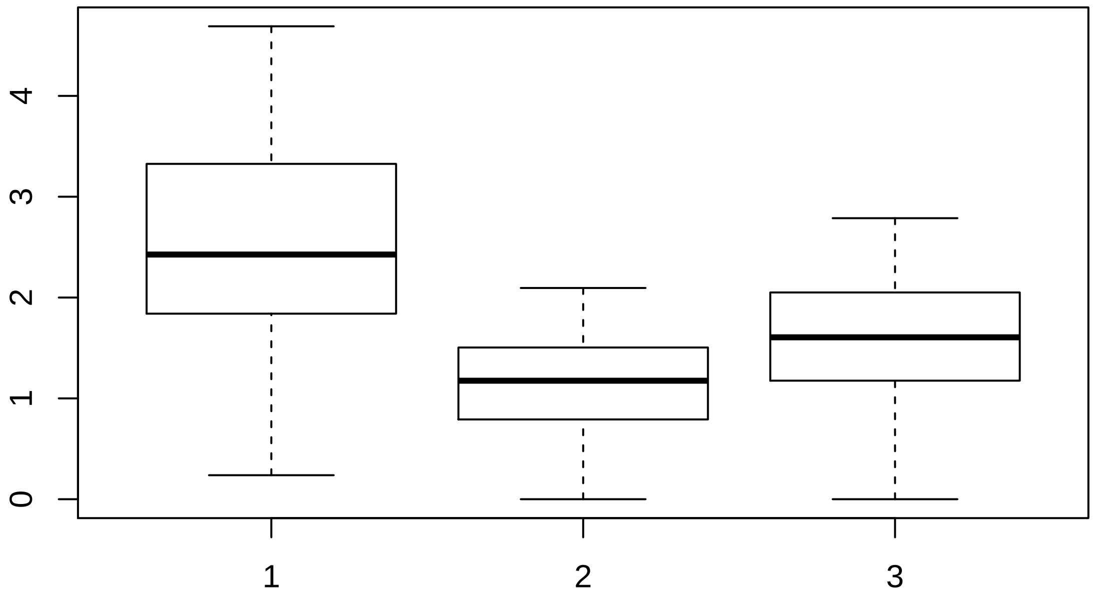


```
#Omega boxplot
PAQ_w <- as.data.frame(PAQ$dN_PAQ/PAQ$dS_PAQ)
PAD_w <- as.data.frame(PAD$dN_PAD/PAD$dS_PAD)
PAF_w <- as.data.frame(PAF$dN_PLF/PAF$dS_PL)
PTV_w <- as.data.frame(PTV$dN_PTV/PTV$dS_PTV)
BlOb_w <- as.data.frame(BlOb$dN_BoOb/BlOb$dS_BoOb)
BlFl_w <-as.data.frame( BlFl$dN_BoFl/BlFl$dS_BoFl)
BlPe_w <- as.data.frame(BlPe$dN_BoPe/BlPe$dS_BoPe)
Bau_w <- as.data.frame(Bau$Baum_dN/Bau$Baum_dS)
Sul_w <- as.data.frame(Sul$Sul_dN/Sul$Sul_dS)

par(mfrow = c(1,1))
boxplot(PAQ_w$`PAQ$dN_PAQ/PAQ$dS_PAQ`,PTV_w$`PTV$dN_PTV/PTV$dS_PTV`,PAD_w$`PAD$dN_PAD/PAD$dS_PAD`,PAF_w$
legend("topleft",title = "Species",c("PAQ","PTV","PAD","PAF","Sul","Bau","BloOb","BlPe","BlFl"),fill=rai
```

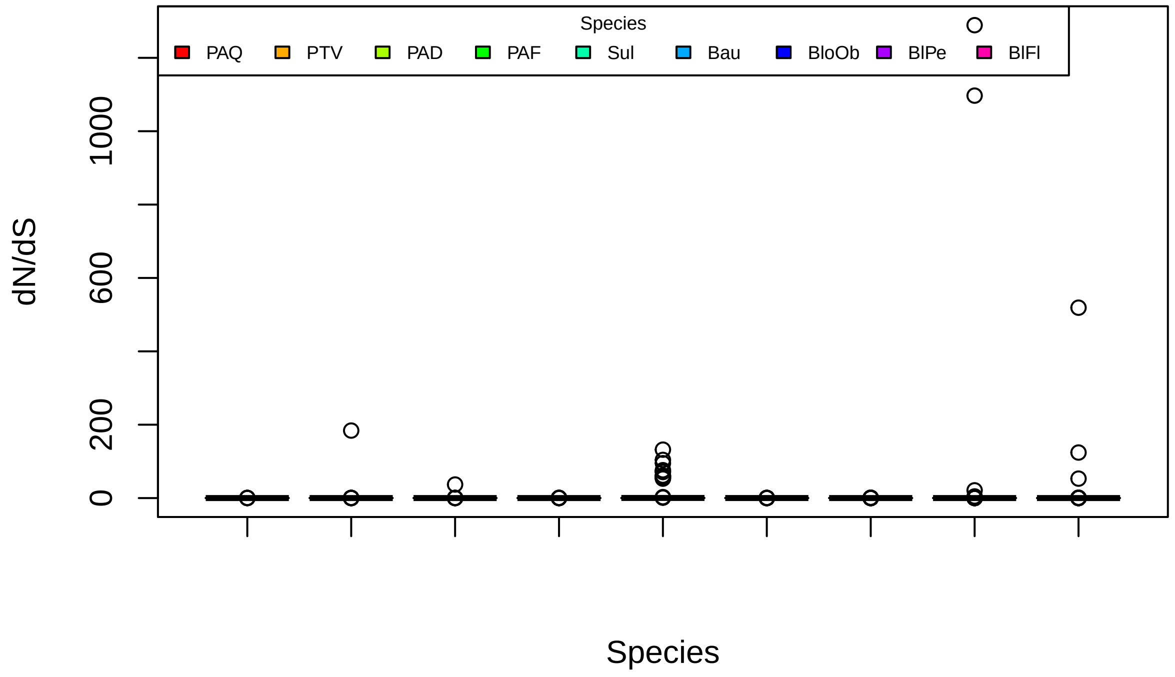


```
#Trim artefactual omega vlaues with 2 as threshold
PAD_w <- subset(PAD_w,PAD_w$`PAD$dN_PAD/PAD$dS_PAD` < 2)
PTV_w <- subset(PTV_w,PTV_w$`PTV$dN_PTV/PTV$dS_PTV` < 2)
Sul_w <- subset(Sul_w,Sul_w$`Sul$Sul_dN/Sul$Sul_dS` < 2)
BlPe_w <- subset(BlPe_w,BlPe_w$`BlPe$dN_BoPe/BlPe$dS_BoPe` < 2)
BlFl_w <- subset(BlFl_w,BlFl_w$`BlFl$dN_BoFl/BlFl$dS_BoFl` < 2)

par(mfrow = c(1,1))
boxplot(PAQ_w$`PAQ$dN_PAQ/PAQ$dS_PAQ`,PTV_w$`PTV$dN_PTV/PTV$dS_PTV`,PAD_w$`PAD$dN_PAD/PAD$dS_PAD`,PAF_w
legend("topleft",title = "Species",c("PAQ","PTV","PAD","PAF","Sul","Bau","BloOb","BlPe","BlFl"),fill=ra
```

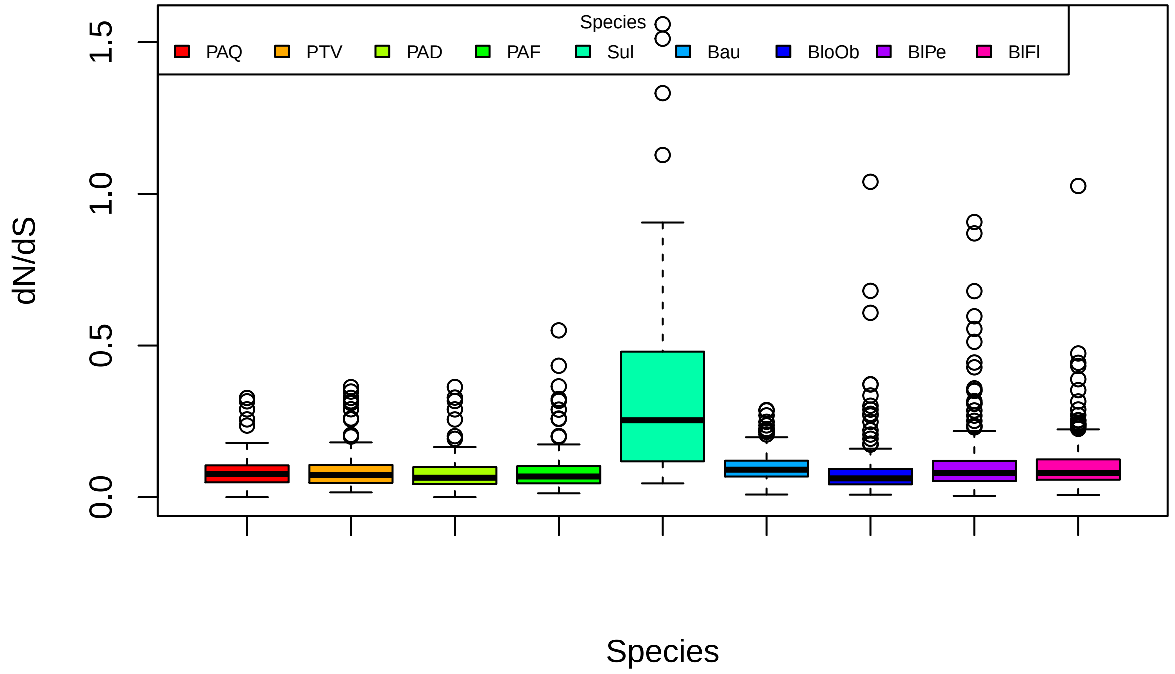

Store the divergence times:

```
ageBtTV <- 90.1002
ageADAF <- 18.339
ageSulcia <- 132.4
ageBaumannia <- 132.4
ageBlOb <- 32.6
ageBlflBlPe <- 18
My <- 1000000
```

Transform dN and dS to subsitution/sites/year (dN/t and dS/t respectively):

```
#By organism
PAQ <- PAQ/(ageBtTV*My)
PTV <- PTV/(ageBtTV*My)
PAD <- PAD/(ageADAF*My)
PAF <- PAF/(ageADAF*My)
BlOb <- BlOb/(ageBlOb*My)
BlFl <- BlFl/(ageBlflBlPe*My)
BlPe <- BlPe/(ageBlflBlPe*My)
Bau <- Bau/(ageBaumannia*My)
Sul <- Sul/(ageSulcia*My)

#Make dataframe with all the symbionts
dN <- c(PAQ$dN_PAQ,PAF$dN_PLF,PTV$dN_PTV,PAD$dN_PAD,BlOb$dN_BoOb,BlFl$dN_BoFl,BlPe$dN_BoPe,Bau$Baum_dN,S
dS <- c(PAQ$dS_PAQ,PAF$dS_PL,PTV$dS_PTV,PAD$dS_PAD,BlOb$dS_BoOb,BlFl$dS_BoFl,BlPe$dS_BoPe,Bau$Baum_dS,Su
Joined <- as.data.frame(cbind(dN,dS))
Joined$symbiont <- as.factor(c(rep("PAQ",183),rep("PAF",183),rep("PTV",183),rep("PAD",183),rep("BlOb",30

#Make a dataframe with Portiera and Sulcia
dN <- c(PAQ$dN_PAQ,PAF$dN_PLF,PTV$dN_PTV,PAD$dN_PAD,Sul$Sul_dN)
dS <- c(PAQ$dS_PAQ,PAF$dS_PL,PTV$dS_PTV,PAD$dS_PAD,Sul$Sul_dS)
Primary <- as.data.frame(cbind(dN,dS))
Primary$symbiont <- as.factor(c(rep("PAQ",183),rep("PAF",183),rep("PTV",183),rep("PAD",183),rep("Sul",1
```

Show the distribution of the data:

```
#Boxplot
par(mfrow = c(1,1))
boxplot(PAQ$dN_PAQ,PTV$dN_PTV,PAD$dN_PAD,PAF$dN_PLF,Sul$Sul_dN,Bau$Baum_dN,BlOb$dN_BoOb,BlPe$dN_BoPe,Bl
legend("topleft",title = "Species",c("PAQ","PTV","PAD","PAF","Sul","Bau","BloOb","BlPe","BlFl"),fill=ra
```

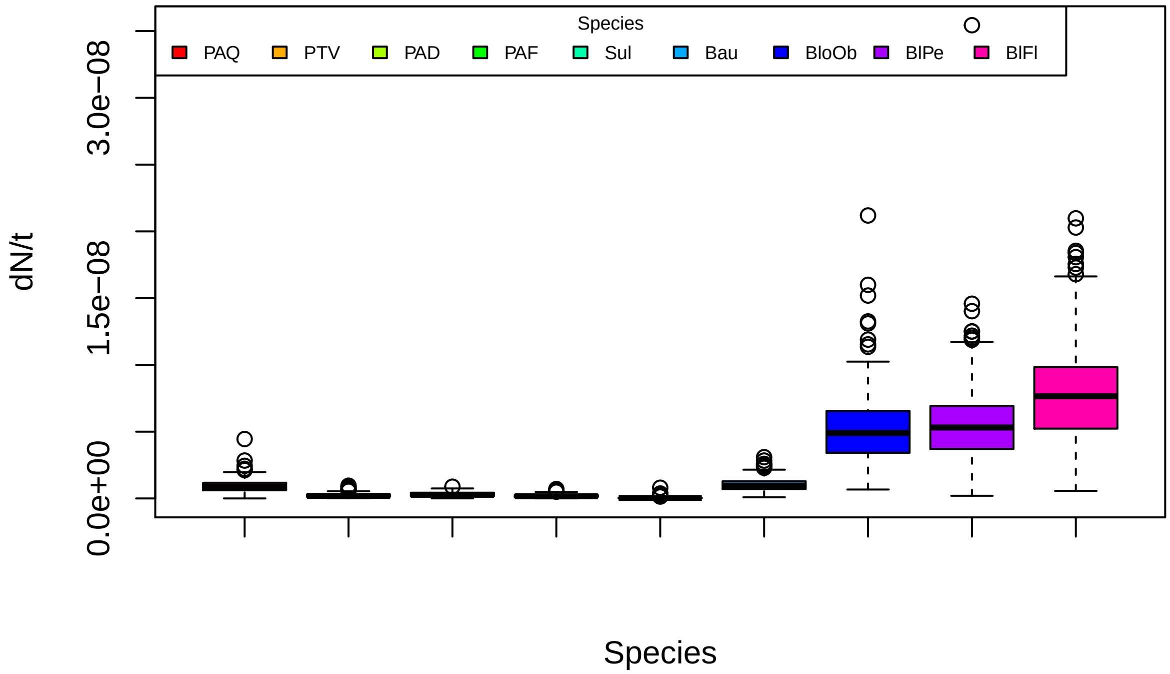


```
boxplot(PAQ$dS_PAQ,PTV$dS_PTV,PAD$dS_PAD,PAF$dS_PL,Sul$Sul_dS,Bau$Baum_dS,BlOb$dS_BoOb,BlPe$dS_BoPe,BlF
legend("topleft",title = "Species",c("PAQ","PTV","PAD","PAF","Sul","Bau","BloOb","BlPe","BlFl"),fill=ra
```

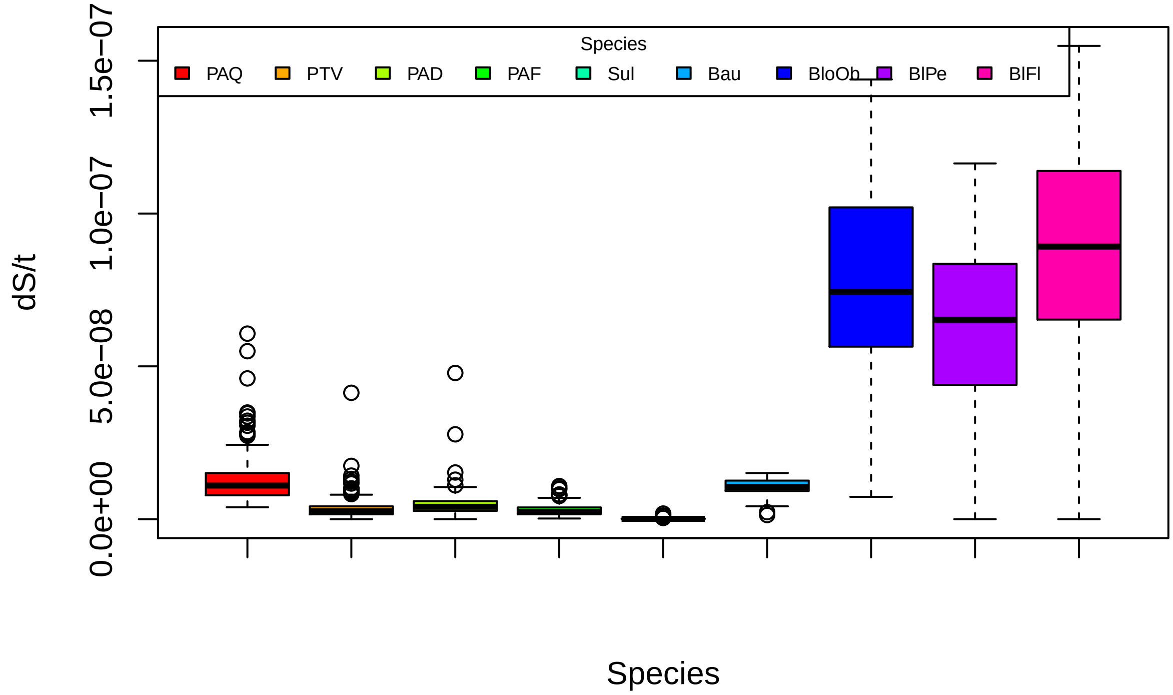

Scatter plot and linear regression for each symbiont:

```
#Portiera Bt
par(cex=0.8)
regPAQ <- lm(PAQ$dS_PAQ ~ PAQ$dN_PAQ)
summary(regPAQ)
```

```
##
## Call:
## lm(formula = PAQ$dS_PAQ ~ PAQ$dN_PAQ)
##
## Residuals:
##        Min         1Q     Median         3Q        Max
## -2.083e-08 -3.705e-09 -1.448e-09  1.562e-09  4.357e-08
##
## Coefficients:
##              Estimate Std. Error t value Pr(>|t|)
## (Intercept) 6.810e-09  1.146e-09   5.943 1.41e-08 ***
## PAQ$dN_PAQ  6.604e+00  1.060e+00   6.232 3.14e-09 ***
## ---
## Signif. codes:  0 '***' 0.001 '**' 0.01 '*' 0.05 '.' 0.1 ' ' 1
##
## Residual standard error: 7.515e-09 on 181 degrees of freedom
## Multiple R-squared:  0.1767, Adjusted R-squared:  0.1721
## F-statistic: 38.84 on 1 and 181 DF,  p-value: 3.144e-09
```

```
plot(x=PAQ$dN_PAQ,y=PAQ$dS_PAQ,col="blue",pch=19,cex=0.5,ylab="dS/t",xlab="dN/t")
abline(regPAQ)
```

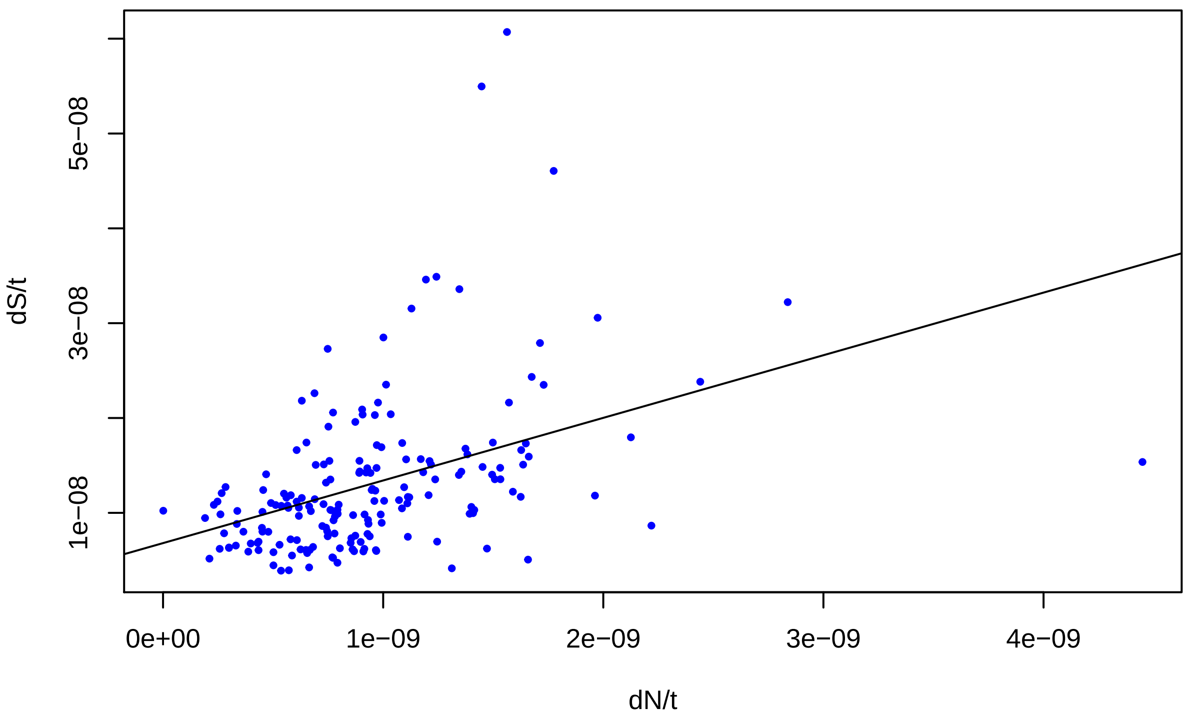


```
#Portiera TV
par(cex=0.8)
regPTV <- lm(PTV$dS_PTV ~ PTV$dN_PTV)
summary(regPTV)
```

```
##
## Call:
## lm(formula = PTV$dS_PTV ~ PTV$dN_PTV)
```

```
##
## Residuals:
##        Min         1Q     Median         3Q        Max
## -1.018e-08 -1.175e-09 -3.388e-10  7.644e-10  2.847e-08
##
## Coefficients:
##              Estimate Std. Error t value Pr(>|t|)
## (Intercept) 2.176e-10  4.326e-10   0.503    0.616
## PTV$dN_PTV  1.434e+01  1.526e+00   9.397   <2e-16 ***
## ---
## Signif. codes:  0 '***' 0.001 '**' 0.01 '*' 0.05 '.' 0.1 ' ' 1
##
## Residual standard error: 3.245e-09 on 181 degrees of freedom
## Multiple R-squared:  0.3279, Adjusted R-squared:  0.3242
## F-statistic: 88.31 on 1 and 181 DF,  p-value: < 2.2e-16
```

```
plot(x=PTV$dN_PTV,y=PTV$dS_PTV,col="blue",pch=19,cex=0.5,ylab="dS/t",xlab="dN/t")
abline(regPTV)
```

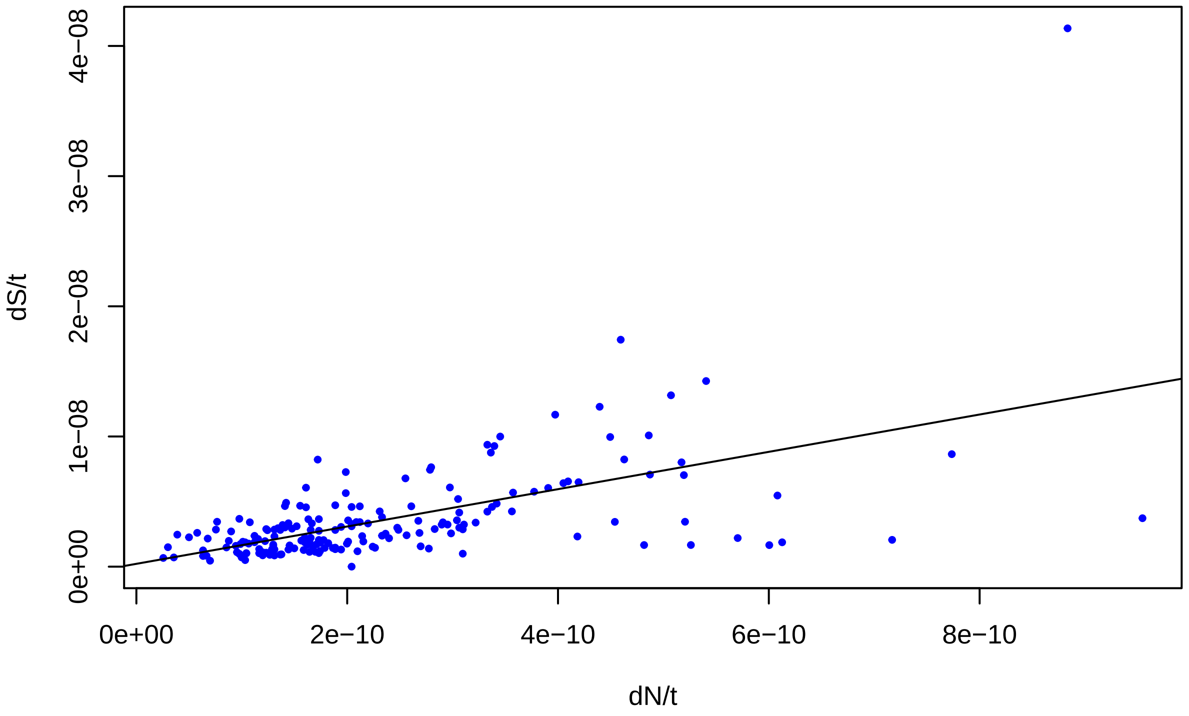


```
#Portiera AD
par(cex=0.8)
regPAD <- lm(PAD$dS_PAD ~ PAD$dN_PAD)
summary(regPAD)
```

```
##
## Call:
## lm(formula = PAD$dS_PAD ~ PAD$dN_PAD)
##
## Residuals:
##        Min         1Q     Median         3Q        Max
```

```
## -5.284e-09 -1.926e-09 -7.160e-10  1.048e-09  4.483e-08
##
## Coefficients:
##                Estimate Std. Error t value Pr(>|t|)
## (Intercept) 2.966e-09  6.282e-10   4.722 4.67e-06 ***
## PAD$dN_PAD  6.047e+00  1.778e+00   3.401 0.000825 ***
## ---
## Signif. codes:  0 '***' 0.001 '**' 0.01 '*' 0.05 '.' 0.1 ' ' 1
##
## Residual standard error: 4.289e-09 on 181 degrees of freedom
## Multiple R-squared:  0.06008,    Adjusted R-squared:  0.05489
## F-statistic: 11.57 on 1 and 181 DF,  p-value: 0.0008252
```

```
plot(x=PAD$dN_PAD,y=PAD$dS_PAD,col="blue",pch=19,cex=0.5,ylab="dS/t",xlab="dN/t")
abline(regPAD)
```

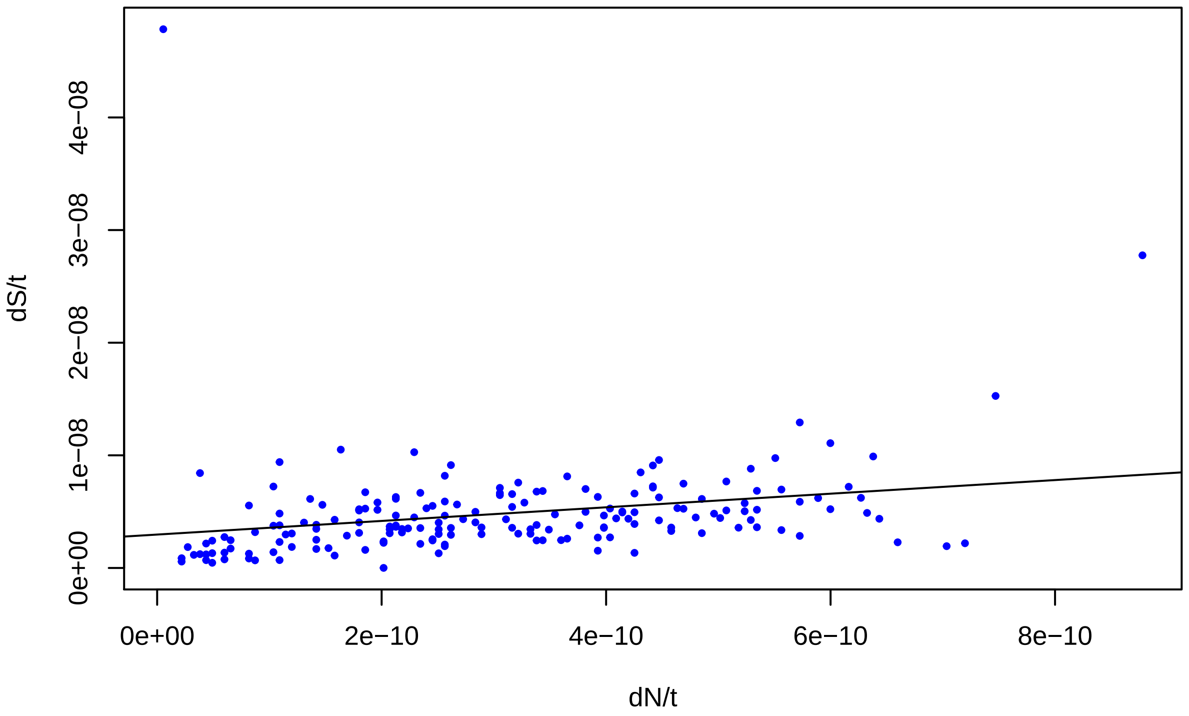


```
#Portiera AF
par(cex=0.8)
regPAF <- lm(PAF$dS_PL ~ PAF$dN_PLF)
summary(regPAF)
```

```
##
## Call:
## lm(formula = PAF$dS_PL ~ PAF$dN_PLF)
##
## Residuals:
##        Min         1Q     Median         3Q        Max
## -3.541e-09 -1.057e-09 -3.715e-10  7.180e-10  7.513e-09
##
## Coefficients:
```

```
##               Estimate Std. Error t value Pr(>|t|)
## (Intercept) 1.554e-09  2.311e-10   6.723 2.25e-10 ***
## PAF$dN_PLF  6.837e+00  9.635e-01   7.096 2.82e-11 ***
## ---
## Signif. codes:  0 '***' 0.001 '**' 0.01 '*' 0.05 '.' 0.1 ' ' 1
##
## Residual standard error: 1.71e-09 on 181 degrees of freedom
## Multiple R-squared:  0.2176, Adjusted R-squared:  0.2133
## F-statistic: 50.35 on 1 and 181 DF,  p-value: 2.816e-11
```

```r
plot(x=PAF$dN_PLF,y=PAF$dS_PL,col="blue",pch=19,cex=0.5,ylab="dS/t",xlab="dN/t")
abline(regPAF)
```

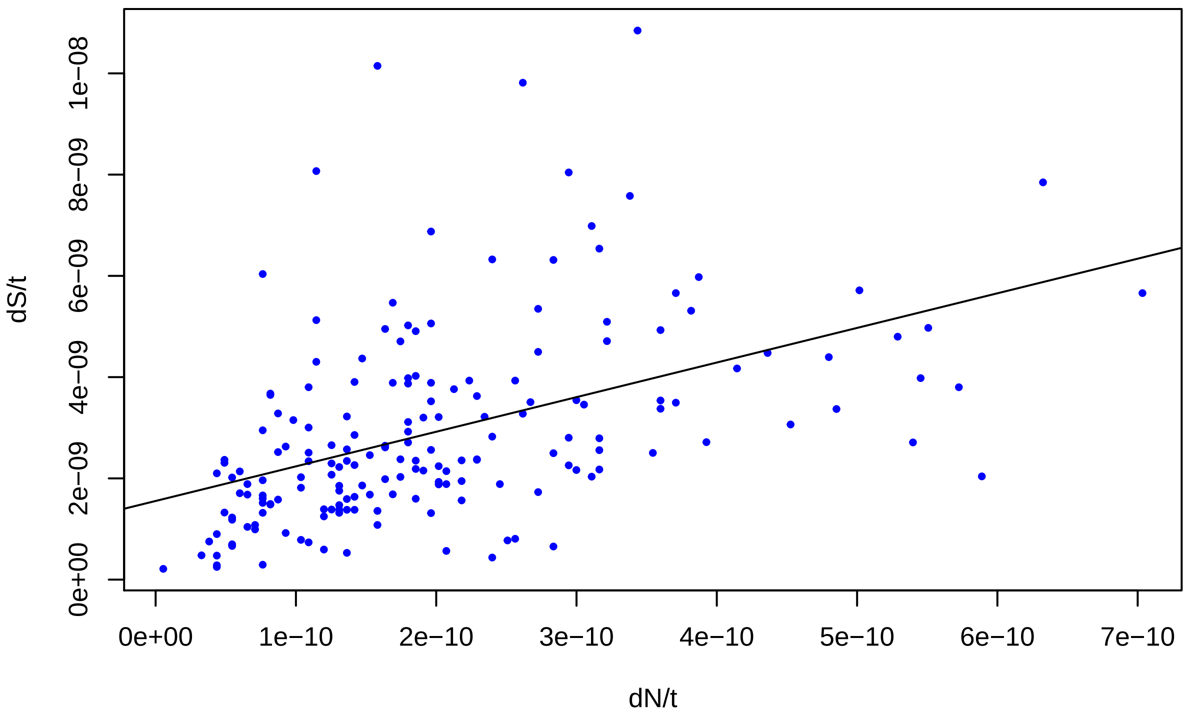


```r
#Sulcia
par(cex=0.8)
regSul <- lm(Sul$Sul_dS ~ Sul$Sul_dN)
summary(regSul)
```

```
##
## Call:
## lm(formula = Sul$Sul_dS ~ Sul$Sul_dN)
##
## Residuals:
##        Min         1Q     Median         3Q        Max
## -4.151e-10 -4.412e-11 -5.500e-13  5.800e-11  3.876e-10
##
## Coefficients:
##               Estimate Std. Error t value Pr(>|t|)
## (Intercept) 3.124e-11  1.278e-11   2.445   0.0161 *
## Sul$Sul_dN  2.071e+00  1.133e-01  18.280   <2e-16 ***
```

```
## ---
## Signif. codes:  0 '***' 0.001 '**' 0.01 '*' 0.05 '.' 0.1 ' ' 1
##
## Residual standard error: 1.162e-10 on 111 degrees of freedom
## Multiple R-squared:  0.7507, Adjusted R-squared:  0.7484
## F-statistic: 334.2 on 1 and 111 DF,  p-value: < 2.2e-16
```

```r
plot(x=Sul$Sul_dN,y=Sul$Sul_dS,col="blue",pch=19,cex=0.5,ylab="dS/t",xlab="dN/t")
abline(regSul)
```

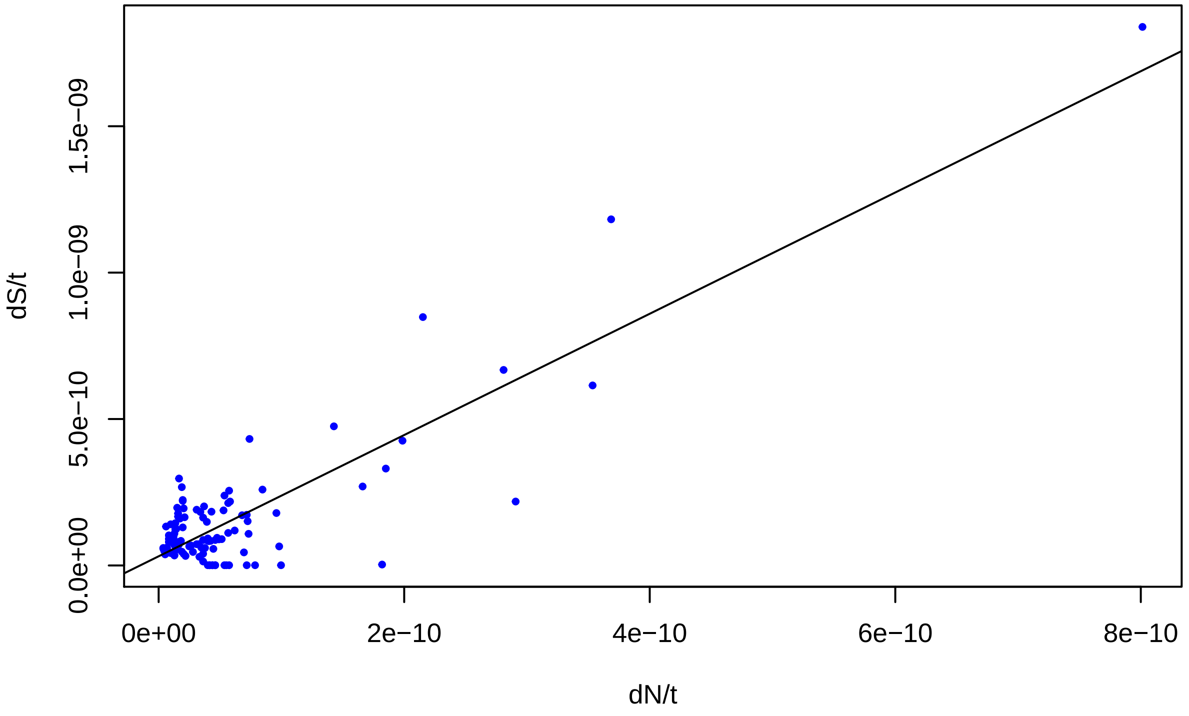


```r
#Baumannia
par(cex=0.8)
regBau <- lm(Bau$Baum_dS ~ Bau$Baum_dN)
summary(regBau)
```

```
##
## Call:
## lm(formula = Bau$Baum_dS ~ Bau$Baum_dN)
##
## Residuals:
##        Min         1Q     Median         3Q        Max
## -8.450e-09 -1.431e-09 -5.680e-11  1.693e-09  4.932e-09
##
## Coefficients:
##              Estimate Std. Error t value Pr(>|t|)
## (Intercept) 9.267e-09  2.572e-10  36.029  < 2e-16 ***
## Bau$Baum_dN 1.398e+00  2.295e-01   6.094 2.38e-09 ***
## ---
## Signif. codes:  0 '***' 0.001 '**' 0.01 '*' 0.05 '.' 0.1 ' ' 1
##
```

```
## Residual standard error: 2.325e-09 on 448 degrees of freedom
## Multiple R-squared:  0.07655,    Adjusted R-squared:  0.07449
## F-statistic: 37.14 on 1 and 448 DF,  p-value: 2.377e-09
```

```r
plot(x=Bau$Baum_dN,y=Bau$Baum_dS,col="blue",pch=19,cex=0.5,ylab="dS/t",xlab="dN/t")
abline(regBau)
```

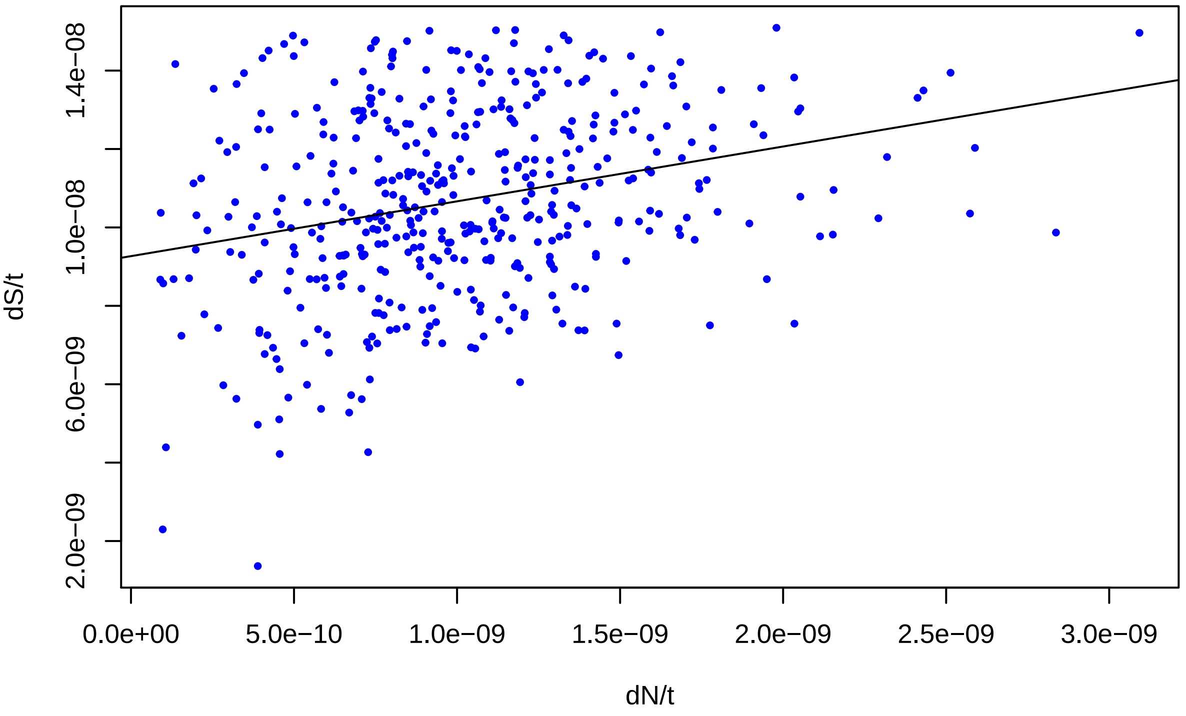


```r
#Blochmannia Ob
par(cex=0.8)
regBlOb <- lm(BlOb$dS_BoOb ~ BlOb$dN_BoOb)
summary(regBlOb)
```

```
##
## Call:
## lm(formula = BlOb$dS_BoOb ~ BlOb$dN_BoOb)
##
## Residuals:
##        Min         1Q     Median         3Q        Max
## -7.474e-08 -2.266e-08 -6.092e-09  2.266e-08  6.686e-08
##
## Coefficients:
##               Estimate Std. Error t value Pr(>|t|)
## (Intercept)  7.263e-08  4.038e-09  17.987   <2e-16 ***
## BlOb$dN_BoOb 1.239e+00  7.001e-01   1.769   0.0778 .
## ---
## Signif. codes:  0 '***' 0.001 '**' 0.01 '*' 0.05 '.' 0.1 ' ' 1
##
## Residual standard error: 3.087e-08 on 303 degrees of freedom
## Multiple R-squared:  0.01023,    Adjusted R-squared:  0.00696
## F-statistic: 3.131 on 1 and 303 DF,  p-value: 0.07784
```

```r
plot(x=BlOb$dN_BoOb,y=BlOb$dS_BoOb,col="blue",pch=19,cex=0.5,ylab="dS/t",xlab="dN/t")
abline(regBlOb)
```

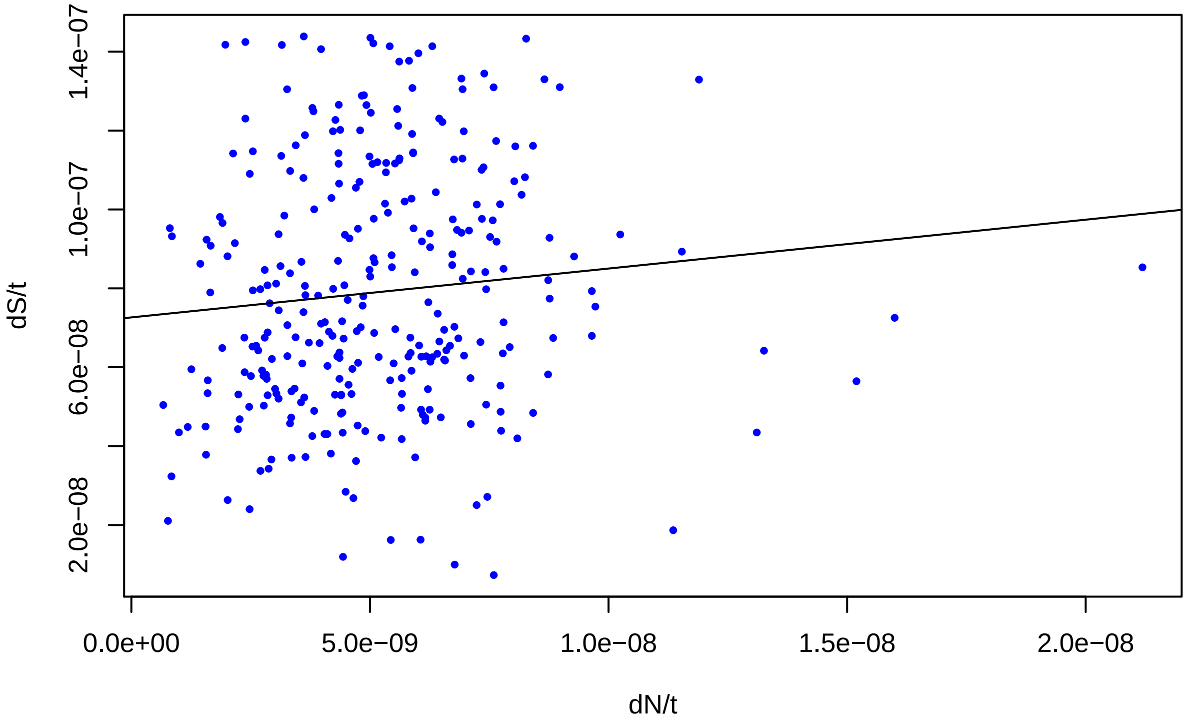


```r
#Blochmannia Pe
par(cex=0.8)
regBlPe <- lm(BlPe$dS_BoPe ~ BlPe$dN_BoPe)
summary(regBlPe)
```

```
##
## Call:
## lm(formula = BlPe$dS_BoPe ~ BlPe$dN_BoPe)
##
## Residuals:
##        Min         1Q     Median         3Q        Max
## -7.098e-08 -1.978e-08  6.620e-10  1.930e-08  5.259e-08
##
## Coefficients:
##               Estimate Std. Error t value Pr(>|t|)
## (Intercept)  5.775e-08  3.192e-09  18.091   <2e-16 ***
## BlPe$dN_BoPe 1.086e+00  4.999e-01   2.173   0.0306 *
## ---
## Signif. codes:  0 '***' 0.001 '**' 0.01 '*' 0.05 '.' 0.1 ' ' 1
##
## Residual standard error: 2.689e-08 on 298 degrees of freedom
## Multiple R-squared:  0.0156, Adjusted R-squared:  0.01229
## F-statistic: 4.721 on 1 and 298 DF,  p-value: 0.03058
```

```r
plot(x=BlPe$dN_BoPe,y=BlPe$dS_BoPe,col="blue",pch=19,cex=0.5,ylab="dS/t",xlab="dN/t")
abline(regBlPe)
```

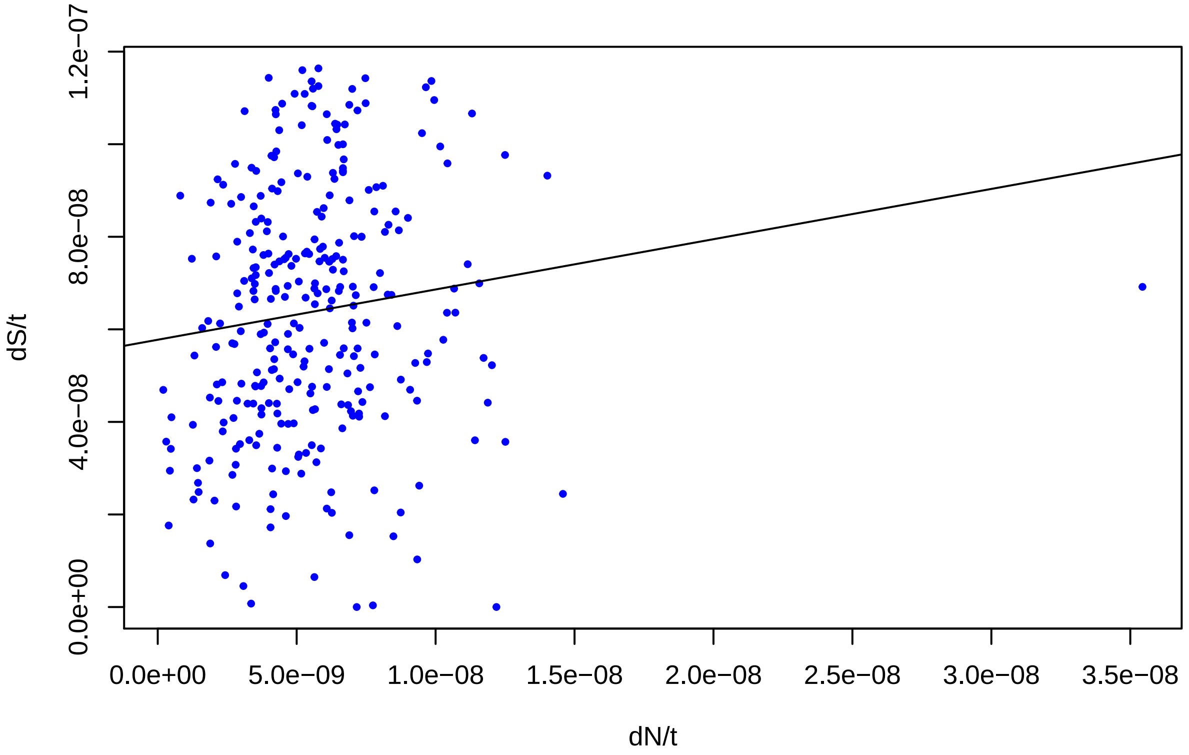


```r
#Blochmannia Fl
par(cex=0.8)
regBlFl <- lm(BlFl$dS_BoFl ~ BlFl$dN_BoFl)
summary(regBlFl)
```

```
##
## Call:
## lm(formula = BlFl$dS_BoFl ~ BlFl$dN_BoFl)
##
## Residuals:
##        Min         1Q     Median         3Q        Max
## -9.094e-08 -2.304e-08 -2.850e-10  2.512e-08  6.642e-08
##
## Coefficients:
##               Estimate Std. Error t value Pr(>|t|)
## (Intercept)  8.692e-08  4.338e-09  20.036   <2e-16 ***
## BlFl$dN_BoFl 3.035e-01  4.938e-01   0.615    0.539
## ---
## Signif. codes:  0 '***' 0.001 '**' 0.01 '*' 0.05 '.' 0.1 ' ' 1
##
## Residual standard error: 3.211e-08 on 300 degrees of freedom
## Multiple R-squared:  0.001258,   Adjusted R-squared:  -0.002071
## F-statistic: 0.3778 on 1 and 300 DF,  p-value: 0.5392
```

```r
plot(x=BlFl$dN_BoFl,y=BlFl$dS_BoFl,col="blue",pch=19,cex=0.5,ylab="dS/t",xlab="dN/t")
abline(regBlFl)
```

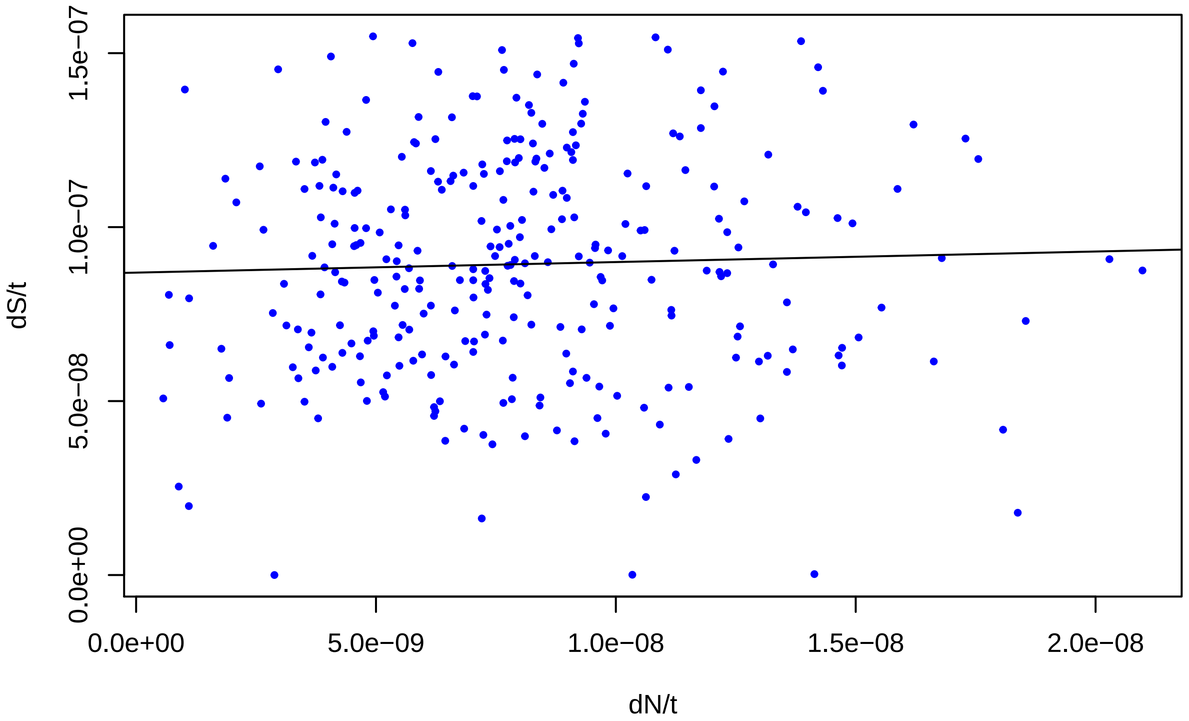

Descriptive statistics for each symbiont:

```r
summary(PAQ)
```

```
##      dN_PAQ               dS_PAQ
##  Min.   :1.110e-12   Min.   :3.901e-09
##  1st Qu.:6.171e-10   1st Qu.:7.799e-09
##  Median :8.735e-10   Median :1.100e-08
##  Mean   :9.458e-10   Mean   :1.306e-08
##  3rd Qu.:1.176e-09   3rd Qu.:1.510e-08
##  Max.   :4.449e-09   Max.   :6.070e-08
```

```r
summary(PTV)
```

```
##      dN_PTV               dS_PTV
##  Min.   :2.553e-11   Min.   :1.110e-12
##  1st Qu.:1.310e-10   1st Qu.:1.607e-09
##  Median :1.887e-10   Median :2.599e-09
##  Mean   :2.360e-10   Mean   :3.601e-09
##  3rd Qu.:3.013e-10   3rd Qu.:4.191e-09
##  Max.   :9.545e-10   Max.   :4.135e-08
```

```r
summary(PAD)
```

```
##      dN_PAD               dS_PAD
##  Min.   :5.453e-12   Min.   :5.450e-12
##  1st Qu.:1.745e-10   1st Qu.:2.707e-09
##  Median :2.726e-10   Median :4.046e-09
##  Mean   :3.050e-10   Mean   :4.810e-09
```

```
## 3rd Qu.:4.280e-10   3rd Qu.:5.889e-09
## Max.   :8.779e-10   Max.   :4.783e-08
```

```r
summary(PAF)
```

```
##      dN_PLF               dS_PL
## Min.   :5.453e-12   Min.   :2.127e-10
## 1st Qu.:1.091e-10   1st Qu.:1.619e-09
## Median :1.745e-10   Median :2.377e-09
## Mean   :2.008e-10   Mean   :2.927e-09
## 3rd Qu.:2.645e-10   3rd Qu.:3.836e-09
## Max.   :7.034e-10   Max.   :1.085e-08
```

```r
summary(Sul)
```

```
##      Sul_dN               Sul_dS
## Min.   :3.776e-12   Min.   :7.553e-13
## 1st Qu.:1.511e-11   1st Qu.:5.211e-11
## Median :3.625e-11   Median :8.837e-11
## Mean   :5.847e-11   Mean   :1.523e-10
## 3rd Qu.:5.665e-11   3rd Qu.:1.775e-10
## Max.   :8.014e-10   Max.   :1.839e-09
```

```r
summary(Bau)
```

```
##     Baum_dN              Baum_dS
## Min.   :8.988e-11   Min.   :1.361e-09
## 1st Qu.:7.090e-10   1st Qu.:9.217e-09
## Median :9.566e-10   Median :1.056e-08
## Mean   :1.014e-09   Mean   :1.068e-08
## 3rd Qu.:1.288e-09   3rd Qu.:1.261e-08
## Max.   :3.093e-09   Max.   :1.509e-08
```

```r
summary(BlOb)
```

```
##     dN_BoOb              dS_BoOb
## Min.   :6.687e-10   Min.   :7.304e-09
## 1st Qu.:3.420e-09   1st Qu.:5.645e-08
## Median :4.902e-09   Median :7.443e-08
## Mean   :5.186e-09   Mean   :7.906e-08
## 3rd Qu.:6.555e-09   3rd Qu.:1.020e-07
## Max.   :2.119e-08   Max.   :1.439e-07
```

```r
summary(BlPe)
```

```
##     dN_BoPe              dS_BoPe
## Min.   :2.000e-10   Min.   :5.560e-12
## 1st Qu.:3.706e-09   1st Qu.:4.396e-08
## Median :5.311e-09   Median :6.527e-08
## Mean   :5.580e-09   Mean   :6.381e-08
## 3rd Qu.:6.914e-09   3rd Qu.:8.340e-08
## Max.   :3.544e-08   Max.   :1.164e-07
```

```r
summary(BlFl)
```

```
##     dN_BoFl              dS_BoFl
##  Min.   :5.667e-10   Min.   :5.560e-12
##  1st Qu.:5.249e-09   1st Qu.:6.534e-08
##  Median :7.656e-09   Median :8.919e-08
##  Mean   :7.949e-09   Mean   :8.933e-08
##  3rd Qu.:9.826e-09   3rd Qu.:1.137e-07
##  Max.   :2.098e-08   Max.   :1.548e-07
```

dN/t and dS/t scatter plots and linear regressions :

```r
#Including Blochmannia and Baumannia lineages:
palette(rainbow(9))
Joined <- subset(Joined,Joined$dN <= 3e-8) #One point deleted for plotting reason
regAll <- lm(Joined$dS ~ Joined$dN )
summary(regAll)
```

```
##
## Call:
## lm(formula = Joined$dS ~ Joined$dN)
##
## Residuals:
##        Min         1Q     Median         3Q        Max
## -1.504e-07 -1.127e-08 -8.366e-09  6.690e-09  1.196e-07
##
## Coefficients:
##              Estimate Std. Error t value Pr(>|t|)
## (Intercept) 1.127e-08  7.597e-10   14.84   <2e-16 ***
## Joined$dN   8.547e+00  1.675e-01   51.02   <2e-16 ***
## ---
## Signif. codes:  0 '***' 0.001 '**' 0.01 '*' 0.05 '.' 0.1 ' ' 1
##
## Residual standard error: 2.737e-08 on 2199 degrees of freedom
## Multiple R-squared:  0.5421, Adjusted R-squared:  0.5419
## F-statistic:  2603 on 1 and 2199 DF,  p-value: < 2.2e-16
```

```r
plot(x=Joined$dN,y=Joined$dS,pch=19,cex=0.5,ylab="dS/t",xlab="dN/t",col=as.factor(Joined$symbiont))
legend("topright",legend=unique(Joined$symbiont),fill=unique(Joined$symbiont))
mtext("R-squared:  0.5421",side=1,line = -4)
abline(regAll)
```

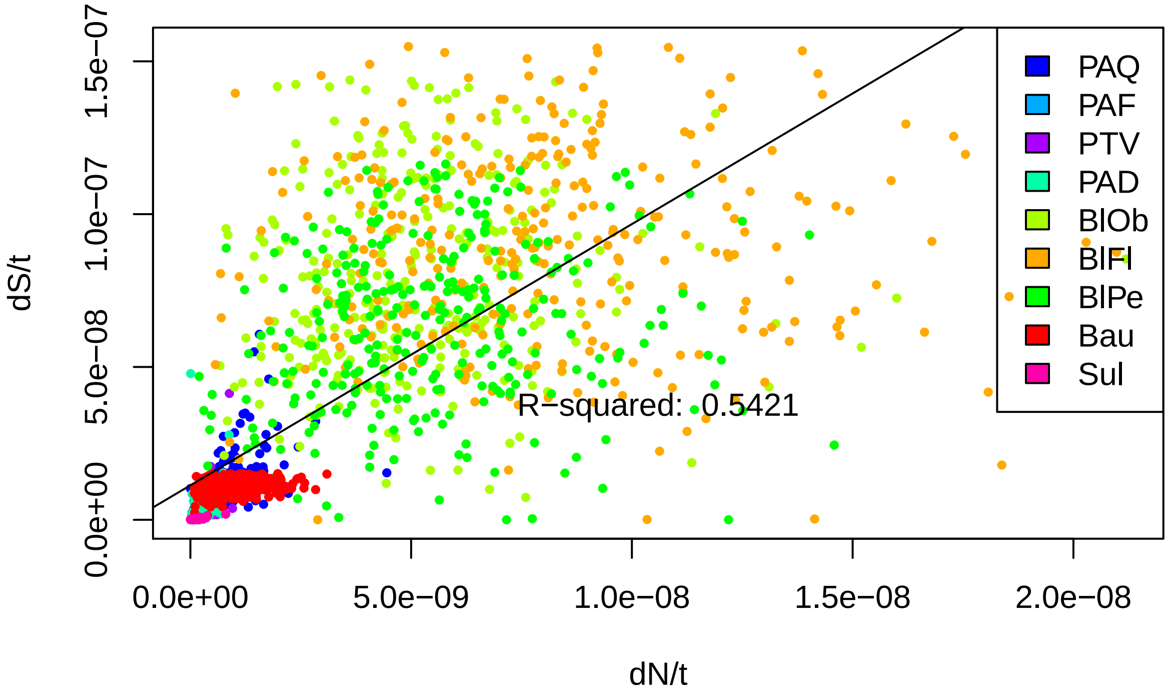


```
#Extreme values trimming
summary(Joined)
```

```
##        dN                   dS                 symbiont
##  Min.   :1.110e-12   Min.   :7.600e-13   Bau     :450
##  1st Qu.:3.217e-10   1st Qu.:4.929e-09   BlOb    :305
##  Median :1.129e-09   Median :1.235e-08   BlFl    :302
##  Mean   :2.904e-09   Mean   :3.610e-08   BlPe    :299
##  3rd Qu.:4.928e-09   3rd Qu.:6.638e-08   PAD     :183
##  Max.   :2.119e-08   Max.   :1.548e-07   PAF     :183
##                                          (Other):479
```

```
boxplot(Joined$dS)
```

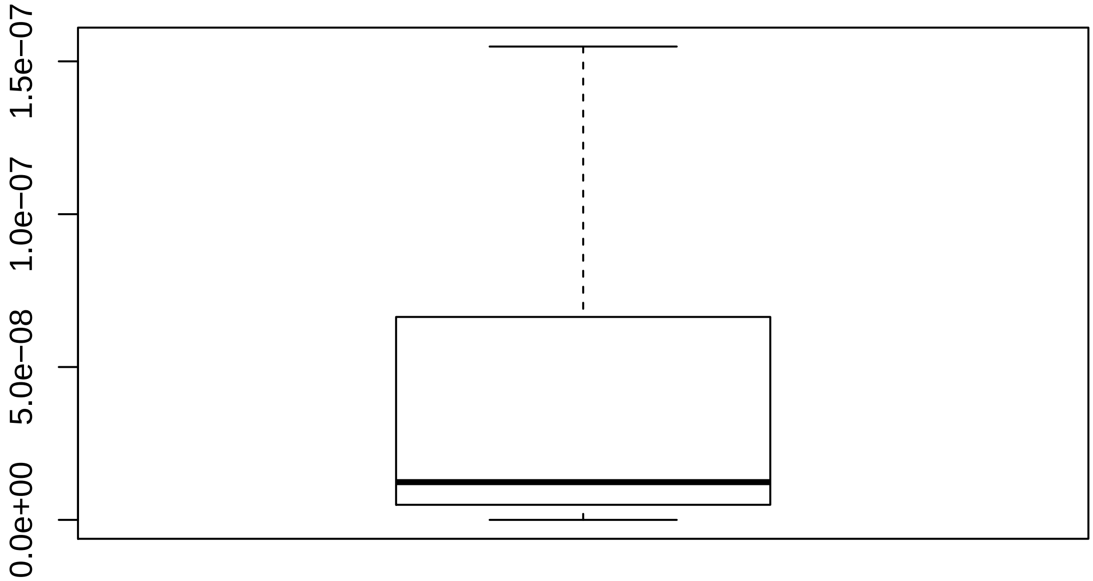


```r
boxplot(Joined$dN)
```

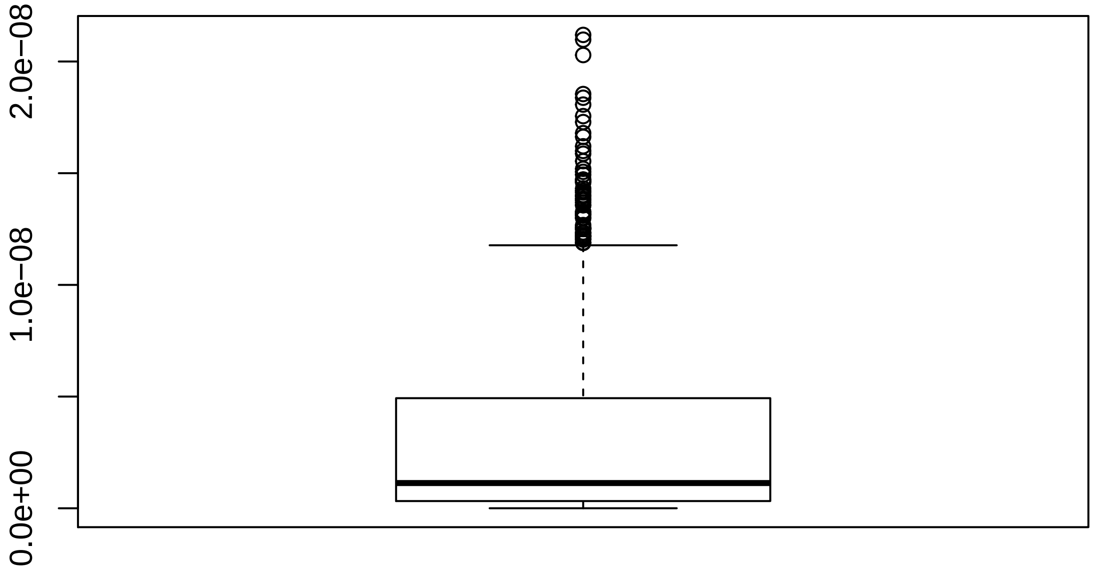


```r
Joined2 <- subset(Joined,Joined$dS <= 6.645e-8)
Joined2 <- subset(Joined,Joined$dN <= 4.936e-9)
boxplot(Joined2$dS)
```

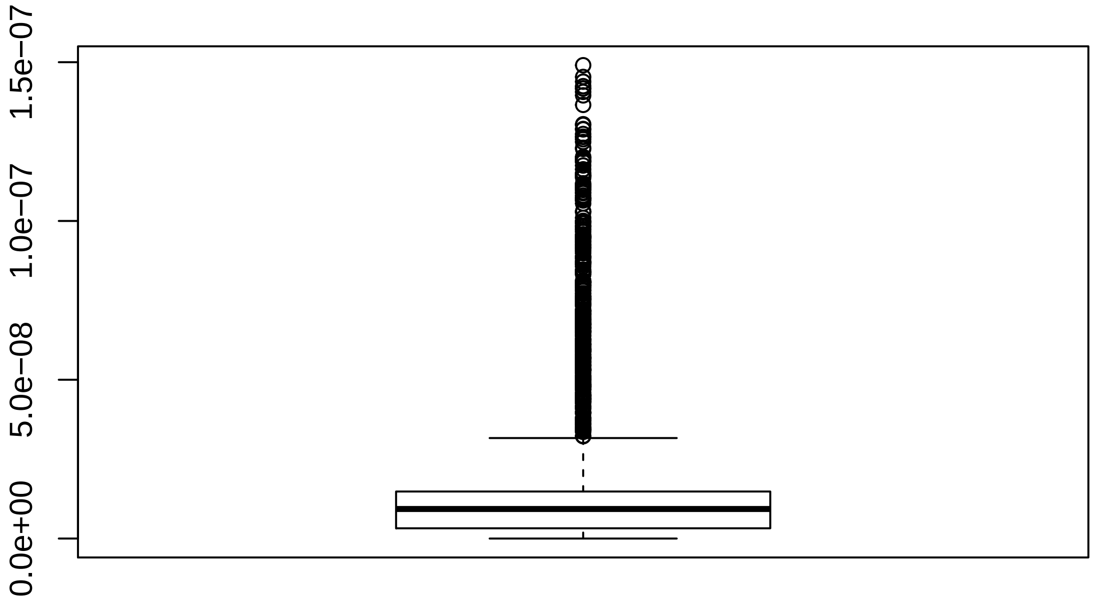


```
boxplot(Joined2$dN)
```

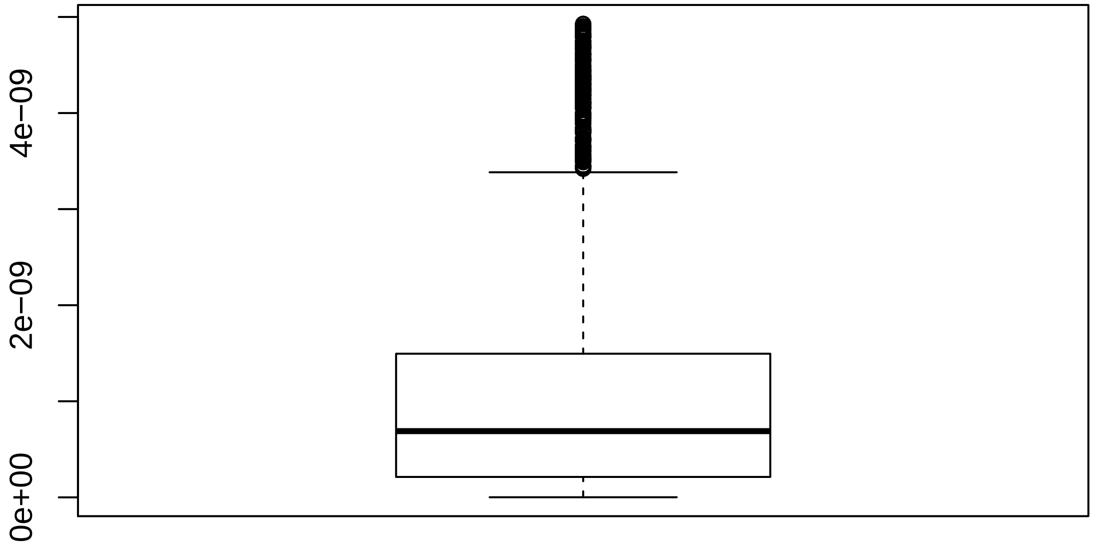


```
regAll <- lm(Joined2$dS ~ Joined2$dN )
summary(Joined2)
```

```
##        dN                  dS                symbiont
##  Min.   :1.110e-12   Min.   :7.600e-13   Bau    :450
##  1st Qu.:2.123e-10   1st Qu.:3.226e-09   PAD    :183
##  Median :6.892e-10   Median :9.301e-09   PAF    :183
##  Mean   :1.187e-09   Mean   :2.078e-08   PAQ    :183
##  3rd Qu.:1.495e-09   3rd Qu.:1.481e-08   PTV    :183
##  Max.   :4.928e-09   Max.   :1.490e-07   BlOb   :154
##                                          (Other):315
```

```r
summary(regAll)
```

```
##
## Call:
## lm(formula = Joined2$dS ~ Joined2$dN)
##
## Residuals:
##        Min         1Q     Median         3Q        Max
## -6.837e-08 -6.237e-09 -3.790e-10  1.976e-09  1.219e-07
##
## Coefficients:
##               Estimate Std. Error t value Pr(>|t|)
## (Intercept) -9.636e-10  5.711e-10  -1.687   0.0917 .
## Joined2$dN   1.832e+01  3.204e-01  57.196   <2e-16 ***
## ---
## Signif. codes:  0 '***' 0.001 '**' 0.01 '*' 0.05 '.' 0.1 ' ' 1
##
## Residual standard error: 1.732e-08 on 1649 degrees of freedom
## Multiple R-squared:  0.6649, Adjusted R-squared:  0.6647
## F-statistic:  3271 on 1 and 1649 DF,  p-value: < 2.2e-16
```

```r
plot(x=Joined2$dN,y=Joined2$dS,pch=19,cex=0.5,ylab="dS/t",xlab="dN/t",col=as.factor(Joined$symbiont))
legend("topright",legend=unique(Joined2$symbiont),fill=unique(Joined2$symbiont))
mtext("R-squared:  0.6649",side=1,line = -4)
abline(regAll)
```

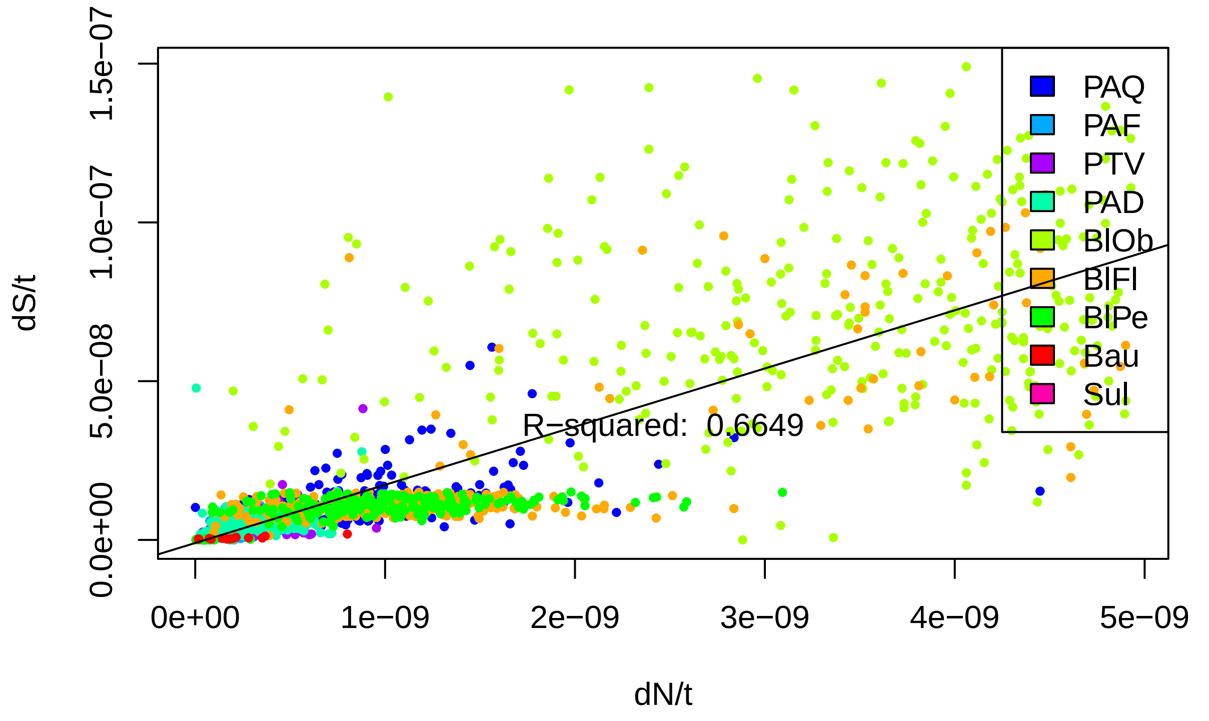


```r
#Only Portiera and Sulcia lineages:
palette(rainbow(5))
regPri <- lm(Primary$dS ~ Primary$dN )
summary(regPri)
```

```
##
## Call:
## lm(formula = Primary$dS ~ Primary$dN)
##
## Residuals:
##        Min         1Q     Median         3Q        Max
## -3.399e-08 -1.680e-09 -9.290e-10  7.920e-10  4.650e-08
##
## Coefficients:
##              Estimate Std. Error t value Pr(>|t|)
## (Intercept) 1.269e-09  2.141e-10   5.928 4.47e-09 ***
## Primary$dN  1.081e+01  3.833e-01  28.192  < 2e-16 ***
## ---
## Signif. codes:  0 '***' 0.001 '**' 0.01 '*' 0.05 '.' 0.1 ' ' 1
##
## Residual standard error: 4.628e-09 on 843 degrees of freedom
## Multiple R-squared:  0.4853, Adjusted R-squared:  0.4847
## F-statistic: 794.8 on 1 and 843 DF,  p-value: < 2.2e-16
```

```
plot(x=Primary$dN,y=Primary$dS,pch=19,cex=0.5,ylab="dS/t",xlab="dN/t",col=as.factor(Primary$symbiont))
legend("topright",legend=unique(Primary$symbiont),fill=unique(Primary$symbiont))
mtext("R-squared:  0.4853",side=1,line = -4)
abline(regPri)
```

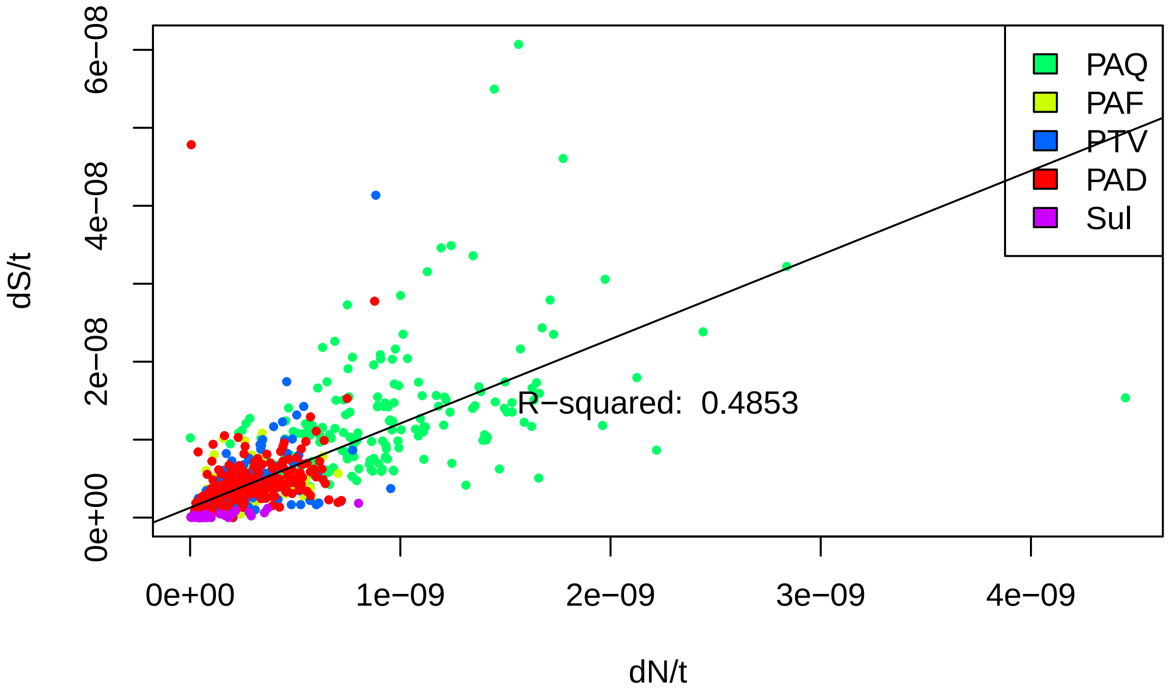


```
#Extreme values trimming
summary(Primary)
```

```
##        dN                   dS              symbiont
##  Min.   :1.110e-12   Min.   :7.600e-13   PAD:183
##  1st Qu.:1.091e-10   1st Qu.:1.494e-09   PAF:183
##  Median :2.290e-10   Median :3.385e-09   PAQ:183
```

```
##  Mean   :3.733e-10   Mean   :5.303e-09   PTV:183
##  3rd Qu.:4.872e-10   3rd Qu.:6.778e-09   Sul:113
##  Max.   :4.449e-09   Max.   :6.070e-08
```

```r
boxplot(Primary$dN)
```

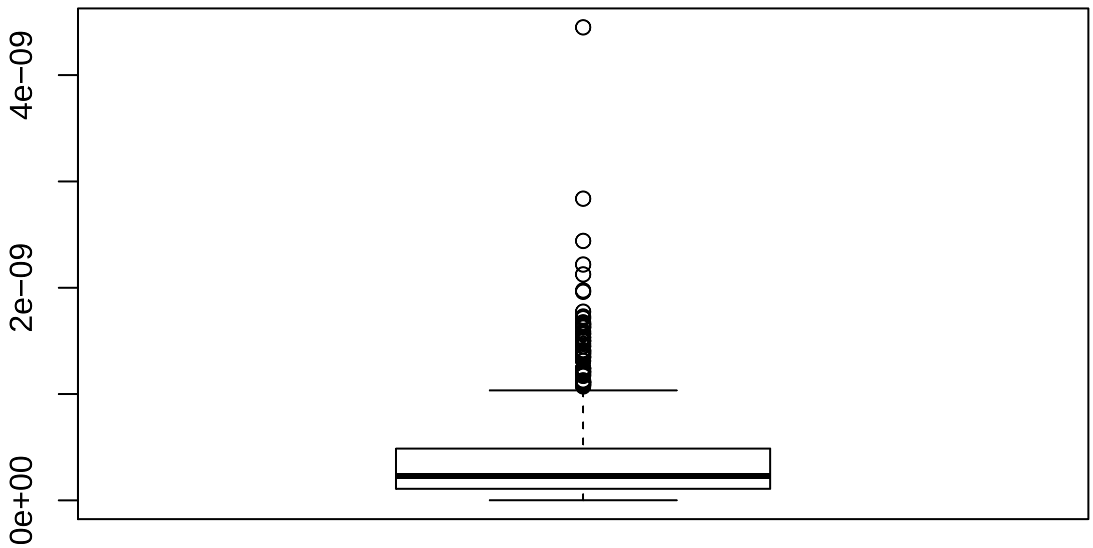


```r
boxplot(Primary$dS)
```

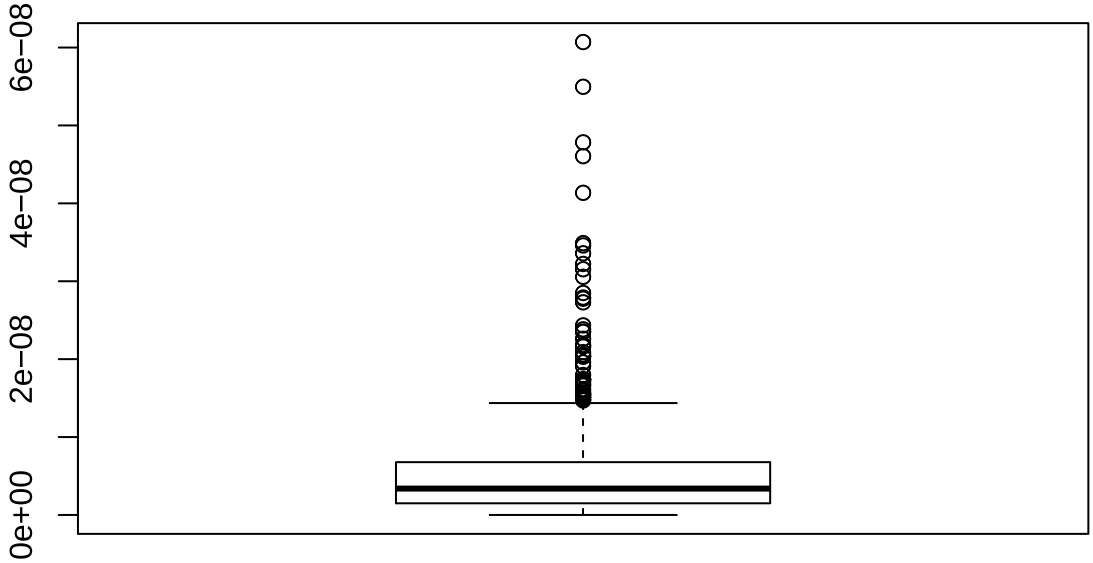


```r
Primary <- subset(Primary,Primary$dS <= 1e-8)
Primary <- subset(Primary,Primary$dN <= 1e-9)
regPri <- lm(Primary$dS ~ Primary$dN )
summary(regPri)
```

```
##
## Call:
## lm(formula = Primary$dS ~ Primary$dN)
```

```
##
## Residuals:
##        Min         1Q     Median         3Q        Max
## -5.879e-09 -1.230e-09 -4.312e-10  9.285e-10  7.342e-09
##
## Coefficients:
##              Estimate Std. Error t value Pr(>|t|)
## (Intercept) 1.167e-09  1.086e-10   10.75   <2e-16 ***
## Primary$dN  8.175e+00  3.325e-01   24.59   <2e-16 ***
## ---
## Signif. codes:  0 '***' 0.001 '**' 0.01 '*' 0.05 '.' 0.1 ' ' 1
##
## Residual standard error: 1.874e-09 on 708 degrees of freedom
## Multiple R-squared:  0.4606, Adjusted R-squared:  0.4598
## F-statistic: 604.5 on 1 and 708 DF,  p-value: < 2.2e-16
```

```
summary(Primary)
```

```
##        dN                  dS             symbiont
##  Min.   :3.776e-12   Min.   :7.550e-13   PAD:176
##  1st Qu.:9.462e-11   1st Qu.:1.250e-09   PAF:181
##  Median :1.898e-10   Median :2.702e-09   PAQ: 64
##  Mean   :2.489e-10   Mean   :3.202e-09   PTV:176
##  3rd Qu.:3.381e-10   3rd Qu.:4.876e-09   Sul:113
##  Max.   :9.933e-10   Max.   :9.994e-09
```

```
plot(x=Primary$dN,y=Primary$dS,pch=19,cex=0.5,ylab="dS/t",xlab="dN/t",col=as.factor(Primary$symbiont))
legend("topright",legend=unique(Primary$symbiont),fill=unique(Primary$symbiont))
mtext("R-squared:  0.4606",side=1,line = -4)
abline(regPri)
```

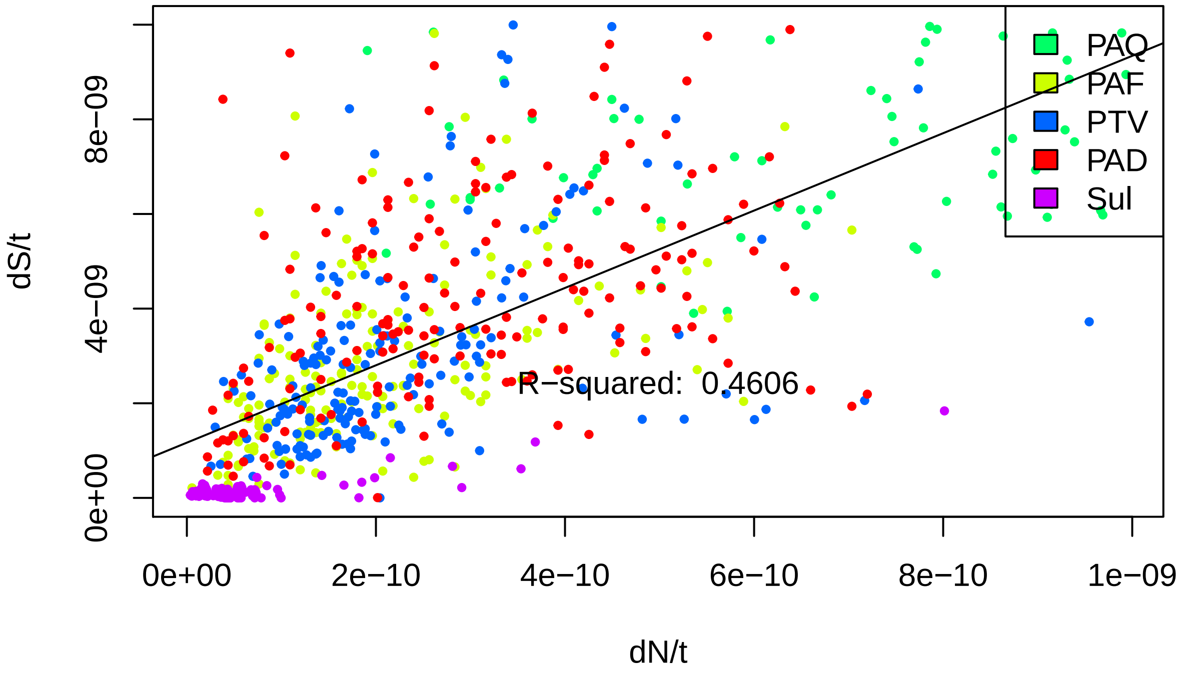

Scatter plot of dN/t and dS/t averages for all lineages:

```r
#Data preparation
options(digits = 15)
sumary <- describeBy(x=Joined,group = Joined$symbiont,digits = 15,mat = TRUE)
symbionts <- as.vector(sumary$group1[c(1:9)])
Averages <- as.data.frame(cbind(sumary$mean[c(1:9)],sumary$mean[c(10:18)]))
colnames(Averages) <- c("average_dN","average_dS")
rownames(Averages) <- symbionts

#Scatterplot plus linear regression
palette(rainbow(9))
regAll <- lm(Averages$average_dS ~ Averages$average_dN )
summary(regAll)
```

```
##
## Call:
## lm(formula = Averages$average_dS ~ Averages$average_dN)
##
## Residuals:
##                 Min                  1Q              Median
## -7.51357741184e-09 -2.63525875823e-09 -6.00668788992e-10
##                  3Q                 Max
##  2.79011621560e-11  1.54945282873e-08
##
## Coefficients:
##                              Estimate        Std. Error  t value
## (Intercept)         1.10879895825e-09 2.93844800668e-09  0.37734
## Averages$average_dN 1.20437720619e+01 7.97357597225e-01 15.10461
##                       Pr(>|t|)
## (Intercept)             0.7171
## Averages$average_dN 1.3402e-06 ***
## ---
## Signif. codes:  0 '***' 0.001 '**' 0.01 '*' 0.05 '.' 0.1 ' ' 1
##
## Residual standard error: 6.74049336629e-09 on 7 degrees of freedom
## Multiple R-squared:  0.970231654493, Adjusted R-squared:  0.965979033706
## F-statistic: 228.149111607 on 1 and 7 DF,  p-value: 1.34018575425e-06
```

```r
plot(Averages$average_dN,Averages$average_dS,col=as.factor(symbionts),pch=19,cex=4,ylab="dS/t",xlab="dN/
legend("top",legend=as.factor(symbionts),fill=as.factor(symbionts),horiz = TRUE,cex=0.6)
mtext("R-squared:  0.9768",side=1,line = -4)
abline(regAll)
```

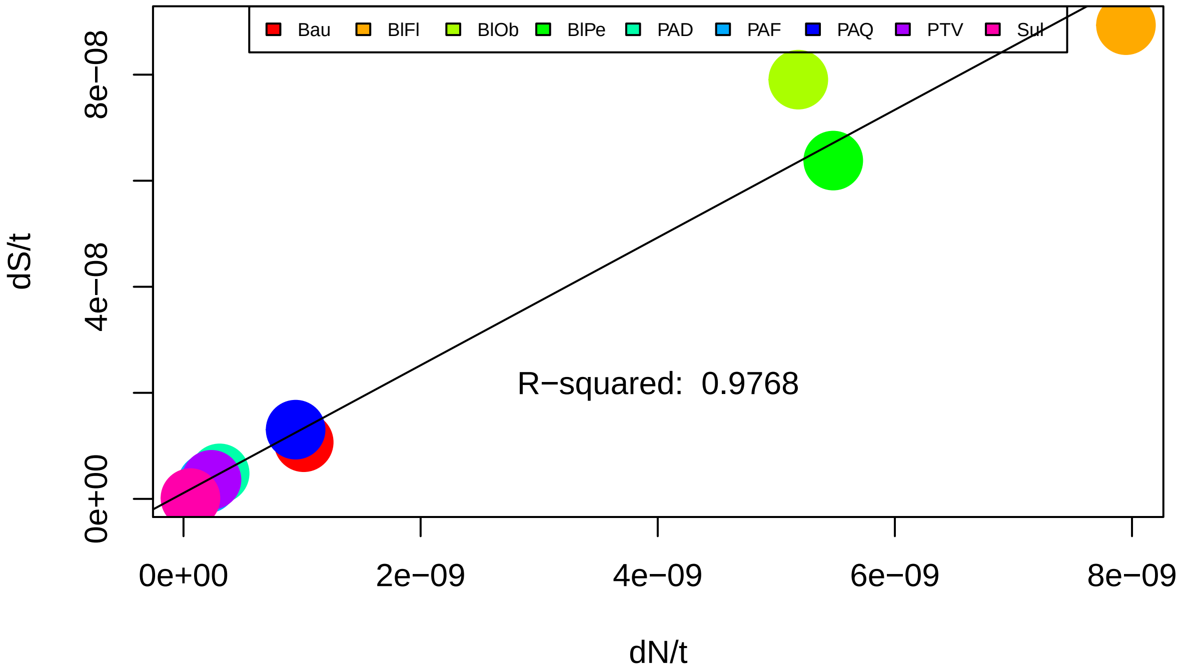

Bau
BlFl
BlOb
BlPe
PAD
PAF
PAQ
PTV
Sul
8e−08
4e−08
0e+00
dS/t
R−squared: 0.9768
0e+00
2e−09
4e−09
6e−09
8e−09
dN/t
